# Associations between parental psychopathology and youth functional emotion regulation brain networks

**DOI:** 10.1016/j.dcn.2024.101476

**Published:** 2024-11-12

**Authors:** Valerie Karl, Dani Beck, Espen Eilertsen, Carmen Morawetz, Thea Wiker, Eira R. Aksnes, Linn.B. Norbom, Lia Ferschmann, Niamh MacSweeney, Irene Voldsbekk, Ole A. Andreassen, Lars T. Westlye, Dylan G. Gee, Haakon Engen, Christian K. Tamnes

**Affiliations:** a PROMENTA Research Center, Department of Psychology, University of Oslo, Oslo, Norway; b Department of Psychology, Yale University, New Haven, United States; c Division of Mental Health and Substance Abuse, Diakonhjemmet Hospital, Oslo, Norway; d Department of Psychology, University of Innsbruck, Innsbruck, Austria; e Department of Psychology, University of Oslo, Oslo, Norway; f Center for Precision Psychiatry, Division of Mental Health and Addiction, University of Oslo & Oslo University Hospital, Oslo, Norway; g KG Jebsen Centre for Neurodevelopmental Disorders, University of Oslo, Oslo, Norway; h Institute of Military Psychiatry Norwegian Armed Forces Joint Medical Services, Oslo, Norway

**Keywords:** Parental psychopathology, Youth, Emotion regulation networks, Functional connectivity, Youth mental health, ABCD

## Abstract

Parental mental health is associated with children’s emotion regulation (ER) and risk for psychopathology. The relationship between parental psychopathology and children’s functional ER networks and whether connectivity patterns mediate the relationship between parent and youth psychopathology remains unexplored. Using resting-state functional magnetic resonance imaging data from the Adolescent Brain Cognitive Development Study (N = 4202, mean age = 10.0) and a multilevel approach, we analyzed the relationship between self-reported parental psychopathology and their offsprings’ connectivity of four ER networks, as well as associations with self-reported youth psychopathology at a 3-year follow-up. Parental internalizing and total problems were associated with 1) higher connectivity between a subcortical-cortical integrative and ventrolateral prefrontal cortical (PFC) network, 2) lower connectivity between dorsolateral and ventrolateral PFC networks involved in cognitive aspects of ER, and 3) lower connectivity within a subcortical ER network (*β* = −0.05–0.04). Parental externalizing and total problems were associated with lower connectivity within the integrative network (*β*_*ext*_ = −0.05; *β*_*tot*_ = −0.04). Mediation analyses yielded direct effects of parental to youth psychopathology, but no mediation effect of ER network connectivity. Overall, our results show that ER network connectivity in youth is related to parental psychopathology, yet do not explain intergenerational transmission of psychopathology.

## Graphical Abstract

**Figure:**
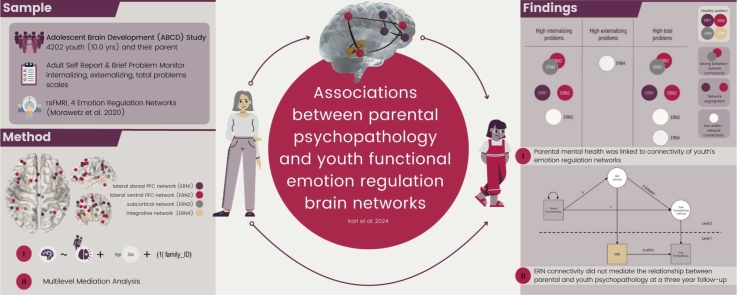

## 1 Introduction

Children of parents with mental health problems have an increased risk of developing mental disorders (Birmaher et al., 2009, Goodman, 2020, Hoffmann and Cerbone, 2002, McLaughlin et al., 2012). This association between parental and youth psychopathology persists independent of genetic confounding (Baldwin et al., 2022, Jami et al., 2021) and thus highlights the role of nurturing factors contributing to this relationship. Parental emotion behavior plays a central role in shaping youth’s development of ER skills (Morris et al., 2007). Considering the co-occurrence of ER difficulties and psychopathology (Aldao et al., 2010), the question arises whether dimensions of current parental psychopathology are associated with youth neural circuitry underlying ER and in turn youth psychopathology. Investigating this relationship has the potential to advance knowledge regarding mechanisms underlying intergenerational transmission of mental health problems.

ER refers to active cognitive processes used to influence one’s own emotional experience and expression (Gross, 1998).The development of these skills is particularly shaped by the emotional climate within parent-child relationships, parents’ reactions towards their child’s emotions, and the child’s observation of the parents’ own regulation skills (Morris et al., 2007, Morris et al., 2017). While emotion regulation skills have the potential to protect against stressors and enhance mental health (Kraiss et al., 2020), emotion dysregulation is a common transdiagnostic characteristic of mental disorders that both predicts and results from psychopathology (Aldao et al., 2010, Lincoln et al., 2022). Consequently, parental psychopathology not only poses a risk for youth through genetic susceptibility, but potentially also by impeding the development of adaptive ER skills (Morris et al., 2007). Several studies have found that youth ER mediates the relationship between parental ER and youth psychopathology (Crespo et al., 2017, Suveg et al., 2011, Tao et al., 2023). A meta-analysis by Lin et al. (2023) provided further evidence that youth ER mediates the association between both parental ER and psychopathology and youth internalizing problems. In a study by Han et al. (2016), parental ER partly accounted for the association between parental psychopathology and youth internalizing (but not externalizing) problems. These studies propose ER as a pathway through which parental psychopathology confers increased risk for psychopathology in their offspring.

Building on this notion, Tan et al. (2020) postulated that parents’ emotion-related behaviors, such as emotion expression or regulation, are associated with youth development of ER skills through changes in neurocircuitry supporting ER. The neural development of emotion regulatory processes involves changes in functional connectivity between limbic and prefrontal regions that reflect adolescents’ increasing ability to involve cognitive strategies in affective processing with age (Caballero et al., 2023, Silvers et al., 2017a, Silvers and Guassi Moreira, 2019). Beyond the recruitment of limbic and prefrontal regions, emotion processing and regulation encompasses a wide network of regions throughout the brain (Karl and Rohe, 2023, Morawetz et al., 2020, Zhang et al., 2019). Based on previously published data on healthy adults, Morawetz et al. (2020) used a meta-analytic approach and identified four large-scale brain networks involved in ER: two cortical networks involved in regulatory processes, one subcortical network associated with emotion reactivity and generation, and one network that integrates cortical and subcortical information. Characterized by strong within-network connectivity and weaker between-network connectivity in healthy adults, divergent connectivity patterns of these networks might reflect disrupted emotion processing and regulation development.

Generally, both emotion-related task-based functional connectivity patterns and resting-state functional connectivity (rsFC) patterns have been associated with psychopathology in children and adolescents (Karcher et al., 2021, Karl et al., 2024, Lees et al., 2021, Voldsbekk et al., 2023, Xia et al., 2018). Moreover, stronger limbic-prefrontal task-based connectivity patterns in youth (12–14 year-olds) have been found to predict ER difficulties after one year, which subsequently correlated with higher psychopathology symptoms after two years (Poon et al., 2022). Considering the beneficial effect of adaptive ER on mental health (Kraiss et al., 2020), typical development of ER circuits is thus crucial to foster ER skills (Casey et al., 2019). Altered functional connectivity between ER-related regions and subsequent ER difficulties might thus contribute to increased risk for psychopathology (Poon et al., 2022).”

Nevertheless, the association between parental psychopathology and youth ER networks and whether youth ER network connectivity in turn relates to youth psychopathology remains unclear. Several studies indicate that psychopathology risk factors that are related to parental psychopathology, e.g., familial risk for depression and anxiety, history of traumatic events, or adverse childhood experiences (Albertina et al., 2022, Barch et al., 2018, Brieant et al., 2021, Herringa et al., 2016, Pawlak et al., 2022), are associated with atypical rsFC patterns of regions involved in ER in middle childhood. Research further shows that parent’s interactions with children is prospectively linked to youth rsFC patterns in emotion circuits (Jiang et al., 2021, Kopala-Sibley et al., 2020).

Few studies have directly addressed the association between parental psychopathology and rsFC patterns in emotion-related regions. Holt-Gosselin et al. (2023) showed that familial high risk of depression was linked to weaker connectivity between the left caudate and cortical regions. On the other hand, Fischer et al. (2022) found that amygdala connectivity with middle frontal and fusiform gyrus differed between youth with a high and low familial risk of mood disorders. Moreover, atypical connectivity patterns between prefrontal and subcortical regions have been identified in offspring of parents with bipolar disorder (Acuff et al., 2019, Singh et al., 2014), major depressive disorder (Chase et al., 2021, Holt-Gosselin et al., 2023), substance use disorder (Kwon et al., 2021), as well as maternal aggression (Callaghan et al., 2017). Taken together, these studies have primarily concentrated on measures indirectly related to parental psychopathology, such as adverse childhood experiences and parenting, or focused on specific diagnoses of parents, leaving potential influences of *current* and *broad* dimensions of psychopathology, such as internalizing, externalizing, and total problem, unexplored.

The aim of the present study was to disentangle the relationship between current parental psychopathology, youth functional ER networks, and consecutive youth psychopathology. Functional ER network connectivity was examined within and between four ER networks (Morawetz et al., 2020). First, we assessed whether parental internalizing, externalizing and total problems were associated with youth ER network functional connectivity. Here, we expected that higher parental psychopathology would be associated with a divergence from typical ER network patterns, i.e., weaker within-network connectivity and stronger between-network connectivity. Further, we predicted that these associations would be particularly pronounced for ER networks associated with cognitive control, i.e., regulation of emotions, reflecting the increasing engagement of cognitive strategies in affective processing and maturation within cognitive control networks from childhood to early adolescence (Luna et al., 2010, Silvers et al., 2017b). The second aim was to test the hypothesis that youth connectivity patterns within and between ER networks partially mediate the relationship between parental internalizing, externalizing, and total problems and later youth internalizing, externalizing, and total problems. The data analysis plans for this study were preregistered on the Open Science Framework (https://osf.io/nwaj8).

## 2 Methods

### 2.1 Sample

We used data from the Adolescent Brain Cognitive Development (ABCD) Study (release 5.0; DOI: 10.15154/8873-zj65), a large, longitudinal dataset following ∼11,800 9–10 year-olds’ development throughout adolescence (Garavan et al., 2018). Recruitment took place between 2016–2018 across 21 different sites in the US with the aim of following the cohort’s neurocognitive and mental health development across ten years. Detailed descriptions of tasks, questionnaires and MRI protocols can be found elsewhere (Casey et al., 2018, Garavan et al., 2018, Volkow et al., 2018). In this study, we used demographic, self-reported parent mental health and neuroimaging data from baseline, and self-reported youth mental health data from the three year follow-up assessment.

Preprocessed tabulated baseline imaging data of N = 5732 participants was obtained from the ABCD-BIDS Community Collection (ABCC) (Feczko et al., 2021), an initiative that pre-processed ABCD Study resting-state functional magnetic resonance imaging (rsfMRI) data to make it openly available and reproducible. We used connectivity matrices based on the Gordon parcellation (Gordon et al., 2016) and subcortical regions (Fischl et al., 2002) to derive ER network (Morawetz et al., 2020) estimates (see 2.4, 2.5 for more details).

Out of the 5732 participants with available connectivity data, 4426 also had complete parental psychopathology data. Although we aimed to avoid arbitrarily selecting one sibling from each family (see Section 2.8.1), we ultimately had to use this method in certain cases to prevent confounding effects and ensure that the assumptions of the statistical model were met. We thus randomly selected one monozygotic twin of 264 participants whose twin was also part of the sample. Out of the remaining 4294, in 91 parental psychopathology was not consistently reported due to different parent or different timepoint for families with two or three siblings. In these cases, we randomly selected one sibling per family for the sample, resulting in a final sample size of 4202 participants (mean age_baseline_ = 10.0, *SD*_baseline_ = 0.6, mean age_follow-up_ = 13.0, *SD*_follow-up_ = 0.6, 50.9 % female). Of the biological parents, 3755 (89.4 %) were biological mothers and 447 (10.6 %) biological fathers. The final sample included data from a total of 3952 families. While the majority (N = 3709) of the families were represented by only one youth, our sample also included data from 236 families with 2 siblings and 7 families with 3 siblings who participated (see Supplemantary Figure S1). Further demographic information can be found in Table 1.

**Table 1:** Sample Characteristics: Age, Sex, Ethnicity, and Family Information.

Age		
	Mean_baseline_	10.0
	*SD*_baseline_	0.6
	Mean_follow-up_	13.0
	SD_follow-up_	0.6
Youth biological sex (N)		
	Female	2140
	Male	2062
Biological parent (N)		
	Mother	3755
	Father	447
Ethnicity (N)		
	Asian	66
	Black	421
	Hispanic	757
	White	2543
	Other	415
Siblings included in sample? (N)		
	No, one child per family in sample	3709
	Yes, two children per family in sample	236
	Yes, three children per family in sample	7

*Note.* Demographics of the sample including information on youth age, sex, ethnicity, and sibling inclusion; N_Total_ = 4202

### 2.2 Adult self report

Parental psychopathology was assessed using the Adult Self Report (ASR), a self-report questionnaire on mental health within the past 6 months that is part of the Achenbach System of Empirically Based Assessment (ASEBA) questionnaires (Achenbach, 2009, Achenbach et al., 2017). One caregiver per participant rated 126 statements about adaptive functioning and problems as “not true (0)”, “somewhat or sometimes true (1)”, or “very true or often true (2)”. Grouping of items yields 8 syndrome scales that can be further summarized to an internalizing problems scale (anxious/depressed, withdrawn, somatic complaints), an externalizing problems scale (aggressive behavior, rule-breaking behavior and Intrusive Behavior), and another problems scale (attention problems, thought problems). In addition, a total problems scale is included as a measure of general psychopathology (Achenbach et al., 2017). To limit the number of analyses and compare parental and youth psychopathology in the same domains, we focused on raw scores from the parent and youth (see below) internalizing, externalizing and total problem scales. Previously evaluated internal consistency for these scales ranged from Cronbach’s alpha of.89 to.97 (Achenbach and Rescorla, 2003). The scales demonstrated good internal consistency, with Cronbach’s alpha of α =.91 (internalizing), α =.85 (externalizing) and α =.95 (Total Problems) in our sample.

### 2.3 Brief problem monitor

For measures of youth psychopathology, we used raw scores on internalizing, externalizing and total problems from the Brief Problem Monitor (BPM; Achenbach, 2009) at a 3-year follow-up visit. Based on items from the Child Behavior Checklist (Achenbach, 2011), this self-report 19-item ASEBA questionnaire is tailored to assess mental health of 6–18-year-olds. Following the same item rating principle as the ASR, the BPM results in internalizing, externalizing, attention, and total problem scales, yet contrary to previously reported satisfactory to high internal consistency (Cronbach’s *α*:.79 −.91) (Piper et al., 2014), the internal consistency at the 3 year-follow up in our sample indicated lower internal consistency (Cronbach’s *α =*.66 for the externalizing scale, Cronbach’s *α =*.79 for the internalizing scale, and Cronbach’s *α =*.85 for the total problems scale.

### 2.4 MRI acquisition and preprocessing

MRI data was acquired across 21 different sites using 31 3-T scanners (Siemens Prisma, General Electric 750, or Phillips) with harmonized scanning parameters (Casey et al., 2018). For the present study we used rsfMRI data collected in 2–4 five-minute scanning sessions, in which the participants were instructed to keep their eyes open and fixate on a crosshair. Details on the imaging acquisition are described in Casey et al. (2018). In brief, rsfMRI data was collected with multiband echo-planar imaging (TR = 800 ms, TE = 30 ms, flip angle = 52°, FOV = 216 mm^2^).

Data were pre-preprocessed by the ABCC team (Feczko et al., 2021) using the ABCD-BIDS pipeline (https://doi.org/10.5281/zenodo.2587210), a modified version of the minimal preprocessing pipeline for the Human Connectome Project (Glasser et al., 2013). Documentation of the pipeline are provided online (https://collection3165.readthedocs.io/en/stable/) and in Feczko et al. (2021). In short, rsfMRI data were de-meaned and de-trended with respect to time, and frames exceeding a frame-wide displacement (FD) threshold of 0.3 mm were filtered out. Next, denoising was applied using a general linear model with signal (mean time series for white matter, cerebrospinal fluid, and global signal), and movement (translational and rotational motion parameters) regressors. Following a band-pass filter between 0.008 and 0.09 Hz using a second-order Butterworth filter, timepoints were censored using an outlier detection approach. Next, the pre-processed data was parcellated using the Gordon 333 ROI atlas (Gordon et al., 2016) and including 19 individualized subcortical parcellations. We used connectivity matrices of 10 min data frames below the FD threshold of 0.2 mm, provided by the ABCC team. The connectivity matrices derived from calculating lag-zero Pearson’ correlation coefficients between all ROIs. Finally, a variance stabilization procedure applied the inverse hyperbolic tangent to the correlation coefficients.

### 2.5 Emotion regulation networks

**Fig. 1:**
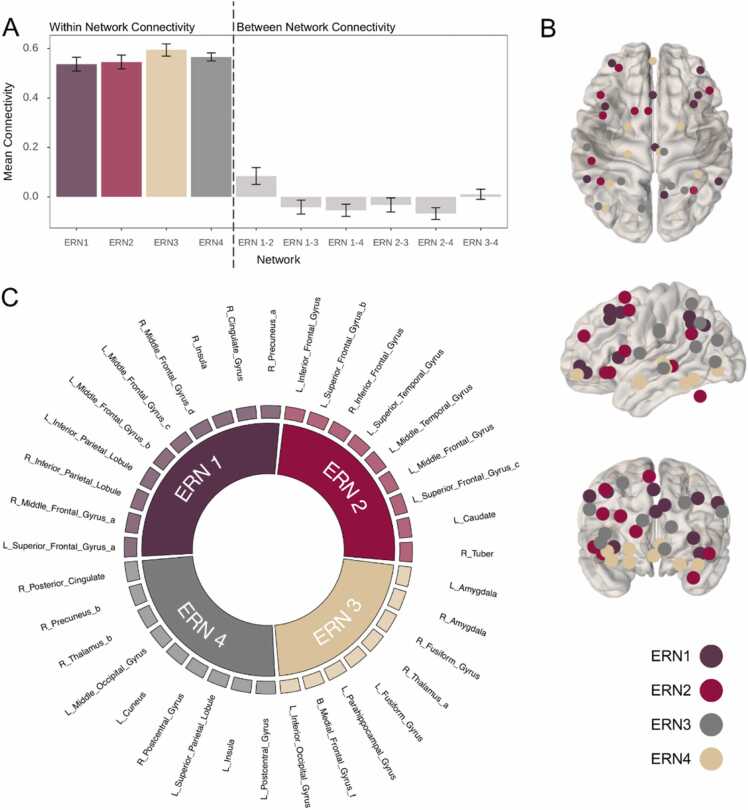
*Emotion Regulation Networks.* A: Mean connectivity within and between each emotion regulation network. Error bars represent +/-1 standard deviation. B and C: Distribution of 36 emotion-regulation regions of interest (ROI). Spheres were created around peak cluster coordinates and based on meta-analytic groupings assigned to one of four emotion-regulation networks as reported in Morawetz et al. (2020). ERN1 (purple) encompasses regions within the left superior frontal gyrus, bilateral middle frontal gyrus and inferior parietal lobus, right insula, cingulate gyrus and precuneus. ERN2 (pink) included bilateral inferior frontal gyrus, left superior frontal and temporal gyrus, left middle frontal and temporal gyrus, left caudate, and right tuber. ERN3 (beige) consists of the bilateral amygdala and fusiform gyrus, and medial prefrontal gyrus, the right thalamus, and left parahippocampal gyrus, and inferior occipital gyrus. ERN 4 (grey) included regions within the bilateral postcentral gyrus, left insula, superior parietal lobule, cuneus, middle occipital gyrus, and the right thalamus, precuneus and posterior cingulate.

Using a meta-analytic grouping approach, Morawetz et al. (2020) identified four large-scale neural networks underlying ER: The first two emotion regulation networks (ERN) were linked to emotion regulatory processes. Both consist of mainly cortical regions, with ERN1 encompassing a fronto-parietal network involving lateral dorsal prefrontal cortical (PFC) regions, and ERN2 comprising ventral PFC regions. ERN3 includes subcortical limbic regions, such as the amygdala and parahippocampus, as well as the ventromedial PFC and has been associated with emotion reactivity and generation. Morawetz et al. proposed that the fourth network, ERN4, acts as an integrative network between the subcortical ERN3 and cortical networks (ERN1 and ERN2). The fourth network has also been linked to the perception of internal sensations related to emotions, emotion generation, and ER. An overview of ROIs within each network and their peak coordinates derived from Morawetz et al. (2020) can be found in Fig. 1 and Supplementary Table S1.

As the preprocessed time-series were in cifti-format, we mapped the ERNs (Morawetz et al., 2020) onto Gordon parcels (Gordon et al., 2016) and subcortical regions (Fischl et al., 2002) before conducting statistical analyses. A detailed description of the procedure can be found in the Supplementary Material (Section 2). In brief, we used nifti versions of the Gordon parcels and subcortical regions in MNI space to avoid issues related to mapping group-average clusters onto cortical surfaces (see Coalson et al., 2018). Using MATLAB (version 9.9.0 R2020b; The MathWorks Inc., 2020) and commands from SPM12 (Ashburner, 2009), we identified voxels of each ERN that overlapped with cortical or subcortical parcels (i.e., we created a mask matrix for each cluster and multiplied it with the matrix containing the corresponding parcel ID for each voxel). The results were saved in a table listing all parcels that mapped onto each ER network and their corresponding number of voxels within that parcel. Some ERNs only comprised of a few voxels or overlapped with the same parcel. We therefore chose to exclude all parcels within an ERN where the number of voxels fell under a threshold of the first quartile (≤ 5 voxels) and assigned each parcel to only one ERN based on the largest voxel overlap. This resulted in a list of parcels for each ER network. Next, we calculated mean correlation coefficients between all parcels within each network as a measure of within-network connectivity, and mean connectivity between all parcel-connections between each network-pairing as a measure of between-network connectivity (Karl et al., 2024, Zhang et al., 2019).

We used two different approaches to derive the within- and between-network connectivity measures. First, we used a “cluster-to-parcel” mapping approach, where we mapped all voxels within an ERN cluster onto each parcel. Second, we constructed 6 mm spheres around the peaks of each cluster (as reported in Morawetz et al., 2020) as an alternative more traditional approach and mapped the voxels within that sphere onto each parcel. We report both sets of results and discuss the different approaches for transparency, but focus on the “peak-to-parcel” approach (see below).

### 2.6 Statistical analyses

All statistical analyses were carried out using R (version 4.2.1; R Core Team, 2022). Prior to the main analyses, we adjusted all variables for scanner effects using neuroComBat (Fortin, 2021), a batch-effect correction tool based on ComBat (Johnson et al., 2007). We included youth age, youth sex, and both parental and youth internalizing, externalizing, and total problems in the model. A comparison of data pre and post harmonization can be found in Figure S2.

For the first part of our analysis, we performed linear mixed effect models with either within- or between-network connectivity as dependent variables, parental psychopathology, youth age and sex as fixed effects, and family ID as a random effect to account for the relatedness in the ABCD sample. We set up one model per psychopathology scale (internalizing, externalizing, total problems) and per network connectivity measure (4 within and 6 between), totaling 30 models. Using the R package *lmeresampler* (version 0.2.4; Loy et al., 2023), we applied bootstrap resampling with 1000 iterations to estimate confidence intervals and bootstrapped *p*-values on the linear mixed effect models. All results were corrected for multiple comparisons using false discovery rate (FDR) correction (Benjamini and Hochberg, 1995) and we set a threshold at *α* = 0.05.

**Fig. 2:**
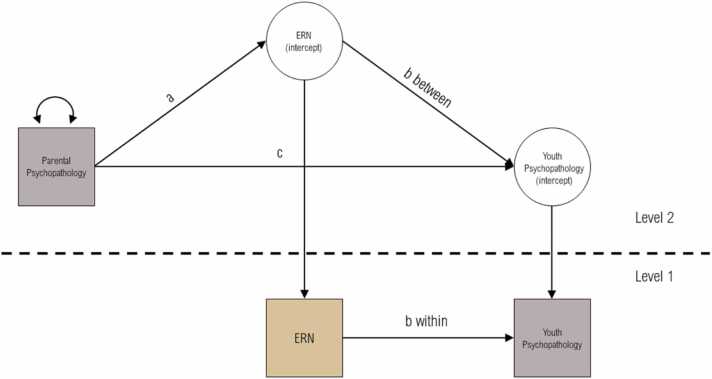
*Multilevel Mediation Model Linking Parental Psychopathology, Youth Emotion Regulation Network Connectivity, and Youth Psychopathology.* Path model of the multilevel mediation model employed to test whether youth functional connectivity of emotion regulation networks mediated the relationship between parental psychopathology at baseline and youth psychopathology at a 3-year follow-up timepoint. Observed variables are presented in squares. Circles represent latent variables. Single-headed arrows represent direct effects and double-headed arrows covariances.

Secondly, we set up multilevel mediation models to test whether the relationship between parental psychopathology and child psychopathology was mediated via connectivity patterns of the child’s functional ER networks (Fig. 2). To test the link between parental psychopathology, ER network connectivity, and youth psychopathology, we employed one mediation model per psychopathology scale and connectivity measure and corrected for multiple comparisons using FDR (Benjamini and Hochberg, 1995). Youth age and sex were included as covariates. In the model specification, children were considered as varying at level-1 and families at level-2. Considering that ER network connectivity and youth psychopathology were observed at level-1, this allowed the relationship between ER network capacity and youth psychopathology to differ within (level-1) and between (level-2) families. These two paths may represent different processes within and between families.

### 2.7 Exploratory analyses

We reran the multi-level model while adjusting for baseline youth psychopathology to see if ERN connectivity mediated the effect of parental psychopathology on youth psychopathology change between baseline and the 3-year follow-up. Furthermore, we specified two multi-level mediation models and compared them by employing a Chi-squared difference test to test whether within and between-family effects were significantly different from each other. In one model we explicitly modelled two different coefficients to estimate the unique relationships between ERN connectivity and youth psychopathology for within and between families, while we did not make this distinction in the second model (see Supplementary Material Section 8). Lastly, we used linear mixed effect models to test whether baseline ERN connectivity was associated with youth psychopathology at the 3-year follow-up. In line with the first part of our main analysis, connectivity measures were treated as the dependent variables, youth psychopathology, youth age (at baseline) and sex as fixed effects, and family ID as a random effect in the model.

### 2.8 Preregistration statement

This study was preregistered on the Open Science Framework (https://osf.io/nwaj8). Deviations from the analysis plan are described below.

#### 2.8.1 Modelling of siblings

Contrary to our preregistration, where we planned to randomly include one sibling per family, we decided to include siblings and employed a linear mixed effects model with family ID as a random effect. The reason for this was that randomly choosing siblings yielded slightly different results, depending on the seed we set for the randomization (see Supplementary Figure S3 for a sample comparison) and the inclusion of siblings would increase statistical power. Moreover, using a multi-level mediation model design permitted to separate the coefficient for the path between network connectivity and youth psychopathology to be split into between and within-family effects. Nonetheless, for cases with inconsistent parent-report (N = 91) and monozygotic twins (N = 264), we randomly included one youth in our sample (see Section 2.1).”

#### 2.8.2 Peak version vs. Cluster version

We found discrepancies between the connectivity measures resulting from the cluster and peak version (see Supplementary Figure S4). We found stronger typical network connectivity patterns for the peak version and therefore proceeded to focus on connectivity derived from the peak-approach. We also decided against recovering Pearson’s correlation coefficients for the analyses using the hyperbolic tangent, and instead used coefficients, to which an inverse hyperbolic tangent had been applied as a variance stabilization procedure.

## 3 Results

### 3.1 Behavioral and demographic results

**Fig. 3:**
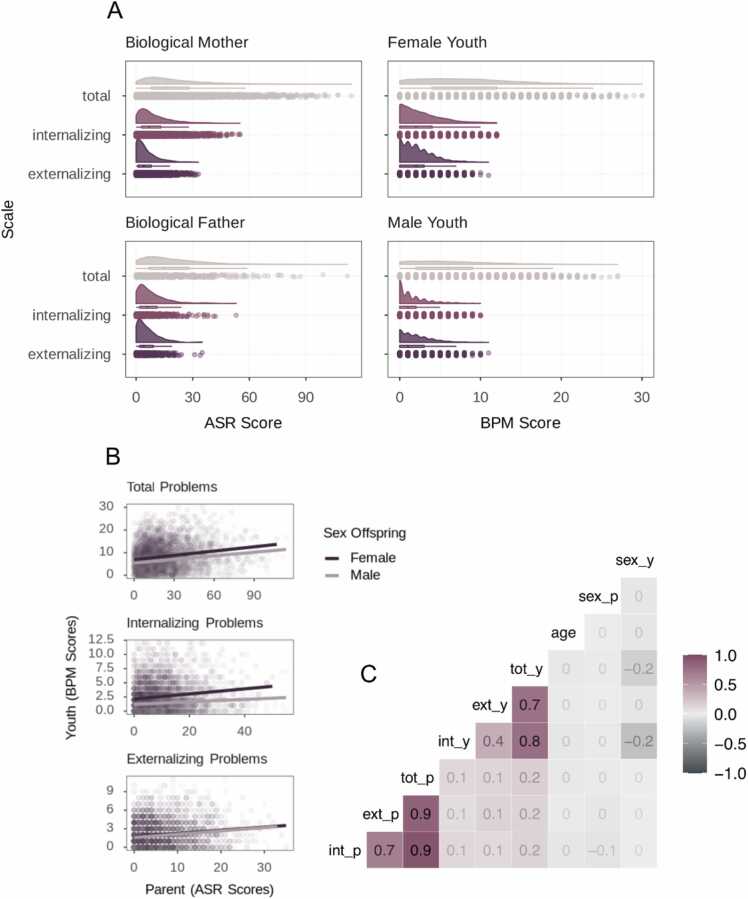
*Youth and Parental Psychopathology Characteristics.* A: Rainplots representing the distribution of internalizing, externalizing, and total problems scores based on sum scores of the Adult Self Report for biological mothers and fathers and the Brief Problem Monitor for female and male youth. B: Associations between parental and youth psychopathology split by offspring sex. C: Pearson correlation coefficients between parental and youth psychopathology dimensions, sex, and youth age. ASR = Adult Self Report; BPM = Brief Problem Monitor; int_p = parental internalizing problems; ext_p = parental externalizing problems; tot_p = parental total problems; int_y = youth internalizing problems, ext_y = youth externalizing problems; tot_y = youth total problems; sex_p = sex parent; sex_y = sex offspring.

An overview of demographic data can be found in Table 1. Correlations between parent-reported parental and youth-reported youth psychopathology are depicted in Fig. 3 and ranged between *r* = 0.1 and *r* = 0.2.

### 3.2 Associations between parental psychopathology and youth FC patterns

**Fig. 4:**
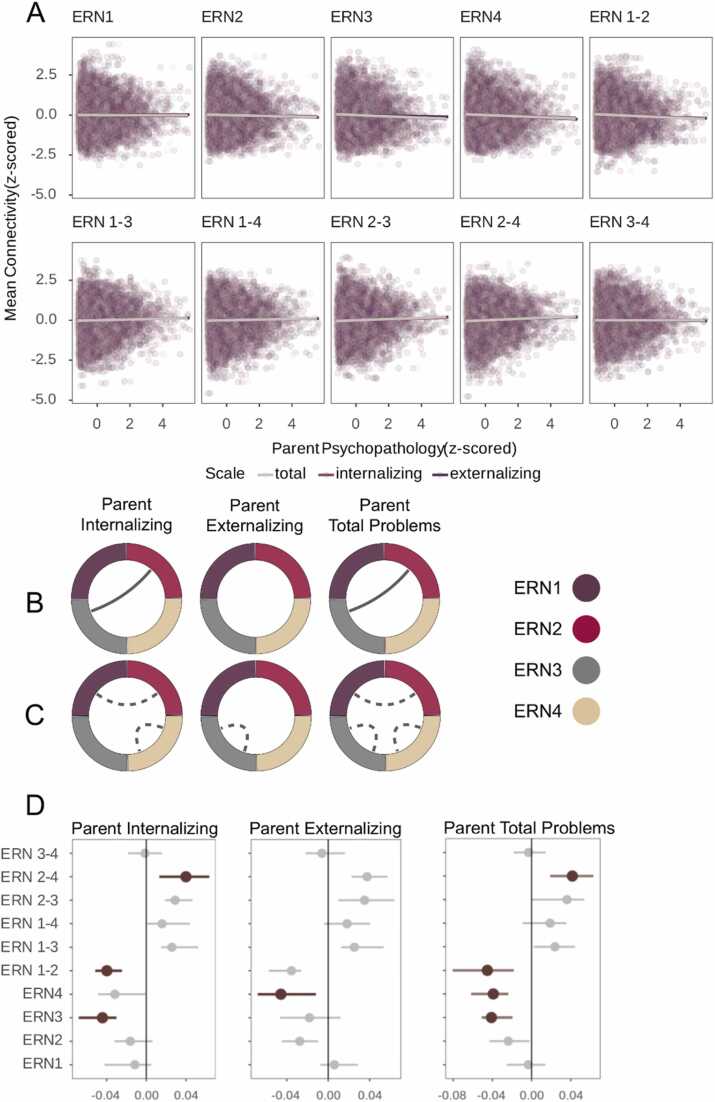
*Associations between Parental Psychopathology and Connectivity of Emotion Regulation Network.* A: Relationships between parental psychopathology dimensions and youth mean functional connectivity (FC) within each emotion regulation (ER). B and C: Significant positive (C) and negative (D) relationships between functional connectivity within and between emotion regulation networks and parental internalizing, externalizing, and total problems. Solid lines indicate positive relationships, and dashed lines negative relationships. D: Bootstrapping results showing 95 % confidence intervals and estimates for each relationship between connectivity measures and psychopathology dimensions. Highlighted dots and lines indicate the association remained significant after correcting for multiple comparisons using FDR. The relationship between total problems and between-connectivity between ERN2 and ERN3 was not significant after FDR-correction (*p*_FDR-corrected_ =.059). Alpha was set to p<.05 in all analyses. ERN = Emotion Regulation Network.

The peak-approach yielded significant negative associations between parental internalizing problems and within-network connectivity in ERN3 (β = −0.04, *p*_*corr*_ =.045) and between-network connectivity between ERN1 and ERN2 (β = −0.04, *p*_*corr*_ =.045), and a significant positive association with between-network connectivity between ERN2 and ERN4 (β = 0.04, *p*_*corr*_ =.045). Parents’ externalizing problems was negatively associated with ERN4 within-network connectivity (β = −0.05, *p*_*corr*_ =.045. Finally, total parental psychopathology was negatively associated with within-network connectivity in ERN3 and ERN4 (β = −0.04, *p*_*corr*_ =.045) and between-network connectivity between ERN1 and ERN2 (β = −0.05, *p*_*corr*_ =.045), and additionally positively associated with between-network connectivity between ERN2 and ERN4 (β = 0.04, *p*_*corr*_ =.045). All results were corrected for multiple comparisons using FDR- correction (Benjamini and Hochberg, 1995) and are presented in Fig. 4 and Table 2. The cluster-approach revealed no significant associations between the parental psychopathology dimensions and connectivity after correcting for multiple comparisons (Table S2).

**Table 2:** Main Effect of Parental Psychopathology on Youth Emotion Regulation Network Connectivity (Peak Version).

Network	*β* estimate	Standard Error	*df*	*T* - value	*p*_*bootstrapped*_	*p*_FDR-corrected_
Internalizing problems						
ERN1	−0.01	0.02	3958.23	−0.75	.474	0.568
ERN2	−0.02	0.02	3934.23	−1.04	.296	0.370
**ERN3**	**−0.04**	**0.02**	**3976.07**	**−2.85**	**.006**	0.045*
ERN4	−0.03	0.02	3931.03	−2.06	.048	0.111
**ERN12**	**−0.04**	**0.02**	**3946.66**	**−2.58**	**.010**	0.045*
ERN13	0.03	0.02	3976.41	1.67	.097	0.182
ERN14	0.02	0.02	3901.02	1.04	.293	0.370
ERN23	0.03	0.02	3961.44	1.88	.064	0.137
**ERN24**	**0.04**	**0.02**	**3949.32**	**2.58**	**.012**	0.045*
ERN34	−0.00	0.02	3979.16	−0.08	.928	0.928
Externalizing problems						
ERN1	0.01	0.02	3862.87	0.39	.696	0.774
ERN2	−0.03	0.02	3821.41	−1.77	.089	0.178
ERN3	−0.02	0.02	3895.15	−1.16	.241	0.328
**ERN4**	**−0.05**	**0.02**	**3806.69**	**−2.97**	**.003**	0.045*
ERN12	−0.04	0.02	3844.41	−2.29	.022	0.060
ERN13	0.03	0.02	3883.47	1.63	.111	0.191
ERN14	0.02	0.02	3760.15	1.18	.238	0.328
ERN23	0.04	0.02	3862.57	2.27	.028	0.070
ERN24	0.04	0.02	3852.39	2.42	.021	0.060
ERN34	−0.01	0.02	3887.37	−0.39	.684	0.774
Total problems						
ERN1	−0.00	0.02	3929.81	−0.21	.828	0.867
ERN2	−0.02	0.02	3900.73	−1.54	.121	0.191
**ERN3**	**−0.04**	**0.02**	**3951.60**	**−2.64**	**.006**	0.045*
**ERN4**	**−0.04**	**0.02**	**3894.21**	**−2.54**	**.011**	0.045*
**ERN12**	**−0.05**	**0.02**	**3915.33**	**−2.93**	**.006**	0.045*
ERN13	0.02	0.02	3949.08	1.53	.117	0.191
ERN14	0.02	0.02	3858.03	1.23	.215	0.322
ERN23	0.04	0.02	3932.61	2.33	.017	0.057
**ERN24**	**0.04**	**0.02**	**3920.46**	**2.68**	**.008**	0.045*
ERN34	−0.00	0.02	3952.06	−0.20	.838	0.867

Note. Main effects of psychopathology on within- and between emotion regulation network connectivity in youth. We employed linear mixed models to test the associations between parental internalizing, externalizing, and total problems and youth connectivity within and between four emotion regulation networks. Youth age and sex were covariates and family ID served as a random effect to account for the nested nature of the data. We used a bootstrapping procedure with 1000 iterations to obtain *p*-values and corrected the results using FDR correction.

ERN = emotion regulation network. * *p* <.05.

### 3.3 Mediation analysis

Multi-level mediation was used to test whether youth ERN connectivity mediated the relationship between basesline parental psychopathology youth psychopathology at a three year follow-up. For both the peak and cluster approach, we found a significant direct effect between parental psychopathology and youth psychopathology in all models, indicating that parental psychopathology was positively associated with youth psychopathology three years later (peak-approach: *β*_*c*_ = [0.12 - 0.22], *p* <.001; cluster-approach: *β*_*c*_ = [0.12 - 0.20], *p* <.001). ERN connectivity was not found to mediate the association between parent psychopathology and later youth psychopathology, as indicated by the absence of a significant indirect effect (see Supplementary Section 6 for full details). Yet total effects significantly explained the relationship between parental psychopathology and youth psychopathology. Due to the nested nature of the data, the analysis yielded two different coefficients between ERN connectivity and youth psychopathology per model, reflecting a between-family and a within-family effect. The resulting path coefficients for within- and between-family effects were not significant after multiple comparison correction using FDR (Benjamini and Hochberg, 1995) and are summarized in Table S5 (peak approach) and Table S6 (cluster approach). These results indicate that ER network connectivity did not mediate the relationship between parental and youth psychopathology.

### 3.4 Exploratory analyses

**Fig. 5:**
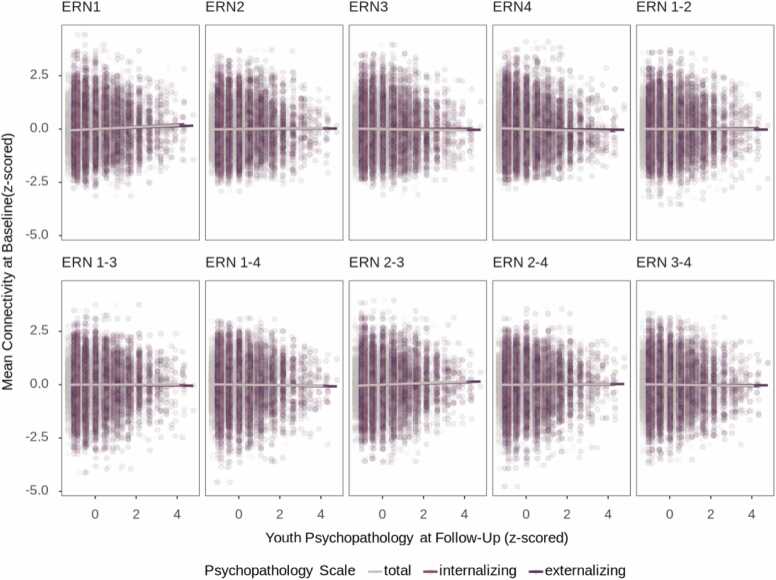
*Associations between Baseline Within and Between Connectivity of Emotion Regulation Networks and Youth Psychopathology at a 3-year Follow-Up.* Associations between mean FC within and between each ER network at baseline and youth’s self-reported internalizing, externalizing, and total problems at a 3-year follow-up.

When including parent-reported youth internalizing, externalizing and total problems at baseline as covariates in our mediation analyses, the results remained unchanged. Associations between youth’s total problems and mean FC of ERNs split into psychopathology risk groups (based on youth’s exposure to parental total problems) are shown in Figure S5. Linear mixed models testing the association between ERN connectivity and youth’s psychopathology yielded a significant small association between youth’s total problems and between-connectivity of ERN2 and ERN3 (*β* = 0.04, *p*_*corr*_ =.049). The associations between ERN connectivity and youth’s psychopathology are presented in Fig. 5 and between ERN connectivity and youth’s emotion regulation skills at the 3-year follow-up in Table S7. Lastly, we conducted follow-up analyses to test the role of parental psychiatric history (described in detail in Supplementary Material Section 12).

## 4 Discussion

In this study we aimed to characterize the relationship between parental psychopathology, youth functional brain ER networks, and youth psychopathology. Parental internalizing problems were significantly associated with weaker within-connectivity in a subcortical network (ERN3), weaker connectivity between the lateral dorsal PFC network (ERN1) and the subcortical network (ERN3), and stronger connectivity between the lateral ventral PFC (ERN2) and integrative (ERN4) network in youth. Parental externalizing problems were linked to weaker within-connectivity in the integrative network (ERN4) in youth. For parental total problems, we found weaker within-connectivity in the subcortical (ERN3), and integrative (ERN4) networks, weaker connectivity between the lateral dorsal (ERN1) and ventral (ERN2) PFC networks, and stronger connectivity between the lateral ventral PFC (ERN2) and the integrative network (ERN4) in youth. Mediation analyses did not support our hypothesis that youth connectivity within and between ER networks would partially mediate the association between parental and later youth psychopathology. Overall, our results show that parental psychopathology co-occurs with subtle disruptions of connectivity patterns within and between ER networks in youth, yet these disruptions do not explain the increased risk for psychopathology in youth.

We did not find that higher parental psychopathology was associated with weaker within-network connectivity in ER networks associated with cognitive control among youth. Instead, parental internalizing and total problems were linked to *greater* network segregation between the two networks encompassing dorsolateral and ventrolateral PFC regions, potentially reflecting more mature and healthy connectivity patterns (Morawetz et al., 2020, Silvers et al., 2017b). These results partially align with Holt-Gosselin et al.'s (2023) finding that maternal depression was linked to weaker connectivity between caudate (ERN2) and left dorsolateral PFC (ERN1) in youth. However, parental internalizing and total problems were positively associated with between-connectivity between the lateral ventral PFC (ERN2) and the integrative (ERN4) networks. These patterns imply that although parental psychopathology is associated with more mature patterns between cognitive networks, the integration of information from prefrontal networks and subcortical regions seems to be disrupted.

Alongside a potentially impaired integration of information from subcortical to cortical regions, our results indicate that biased processing of emotions could already start in early stages of emotion processing that involves the perception of emotional stimuli. Parental externalizing and total problems were negatively related to within-network connectivity in the integrative network (ERN4), and with internalizing and total problems in the subcortical network (ERN3). Regions within both networks are involved in emotion generation and their development might be prone to environmental influences, such as family factors (Zhi et al., 2023). Eisenberg et al. (1998) postulated that effects of parental emotion behavior on a child's processing and regulation of emotions are mediated through arousal, which according to Tan et al. (2020) operates via structural and functional emotion-related networks. The integrative network (ERN4) has been associated with interoception, i.e., the perception and interpretation of body signals such as a heighted heart rate, and the integration of this information on arousal with cortical regions (Morawetz et al., 2020). Arousal can affect cohesions within networks associated with salience detection (Young et al., 2017) and can enhance the processing of salient cues (Sutherland and Mather, 2018). Yet, it has also been linked to neural underpinnings of psychopathology (Koenig et al., 2016, Mulcahy et al., 2019). Therefore, weaker connectivity within the integrative network (ERN4) might stem from altered saliency detection as a result of dimensions of parental psychopathology. Among youth, altered connectivity patterns between regions involved in interoception and salience detection could indicate biased arousal perception and interpretation that stems from adaptations to different life experiences, or genetic predispositions associated with parental psychopathology.

Aligning with previous research linking lower volume and connectivity between limbic regions and parental internalizing problems (Albar and Sattar, 2022, Singh et al., 2018), youth connectivity within the subcortical network was associated with parental internalizing and total problems. This finding further indicates that parental psychopathology is associated with neural circuits underlying emotion generation in youth. Emotion regulation strategies have different outcomes depending on whether an emotion was generated “bottom-up”, i.e. through emotion-relevant perceptual aspects of a stimulus, or “top-down”, i.e. via cognitive appraisal. While reappraisal could successfully regulate top-down generated emotions, this ER strategy can have an opposite effect on bottom-up generated emotions (McRae et al., 2012). Both mechanisms are linked to distinct neural processes that both involve regions within the subcortical network (Ochsner et al., 2009). This ER network is associated with emotion perception and generation (Morawetz et al., 2020), thus alterations within this network could negatively impact the generation and subsequent regulation of emotions.

Together, these findings show that parental psychopathology is associated with youth connectivity patterns within and between ER networks. However, even though the mediation analyses highlighted direct effects between parental and youth psychopathology three years later, youth connectivity patterns within ER networks did not mediate this relationship. Interestingly, the data suggest that the direction of the relationships between ER network connectivity and youth psychopathology may vary across between-family and within-family effects. The differences between the two estimates were not significant after applying FDR-correction. Yet, considering that siblings represented a minority in our sample, our design might entail power issues that impede a thorough examination of within-family effects. A between-family effect would include e.g., culture and socioeconomic factors, the within-family estimate reflects remaining relationship between network connectivity and youth psychopathology after accounting for siblings’ shared environmental and genetic factors. We also observed that our initial approach of selecting one sibling per family at random led to varying results, influenced by the specific randomization seed used. Consequently, we suggest that future studies might benefit from employing statistical approaches that avoid random selection of siblings and instead allow for the exploration of both between-family and within-family effects.

Although the large sample size and multilevel approach are strengths of the current study, our methodological choices also yield important limitations to consider when interpreting our results. First, the current study focused on current and broad parental psychopathology within the last 6 months. Our results complement previous neuroimaging studies on familial risk that have primarily used group designs and measures on parental history of specific diagnoses (e.g. Fischer et al., 2022; Holt-Gosselin et al., 2023). While our results show a connection between current parent and youth psychopathology, environmental effects on a child’s brain development might be stronger in earlier developmental stages (e.g. Ferschmann et al., 2024; Kopala-Sibley et al., 2020; Posner et al., 2016) and are linked to brain maturation during adolescence (Beck et al., 2024). Given the complex interplay of parental and youth psychopathology with youth’s ER development and the limitations of our study design, we cannot conclude that associations with ER circuitry are driven by parental instead of youth psychopathology. Together with findings highlighting connectivity changes around the transition to adolescence (Gabard-Durnam et al., 2014, Gee et al., 2013), around the mean age of our sample, longitudinal data with both task-based and resting state fMRI data are needed to better understand the relationship between parental psychopathology and youth development of ER networks. Furthermore, we decided to include both biological fathers and mothers in our study. However, previous research has highlighted a potential moderating effect of sex from both parent and child on the relationship patterns between ER and psychopathology (Holt-Gosselin et al., 2023, Itahashi et al., 2020). Moreover, it is important to note that our methodological approach of mapping task-based clusters and peak spheres onto Gordon parcels can lead to overlap discrepancies. As depicted in Figure S4, both approaches (and specifically the peak approach) led to typical within and between network connectivity patterns. Lastly, this study utilized self-report measures from one biological parent and their youth, which potentially biases our findings (Sourander et al., 1999, Youngstrom et al., 2003). The poor reliability of the youth’s psychopathology scales additionally constrains the generalizability of our results. We were not able to properly control for youth psychopathology when assessing the associations between parental psychopathology and ER network connectivity, as there is no youth-reported psychopathology data available from baseline. Future studies should thus carefully consider potential discrepancies between parent and youth-reported youth psychopathology, and also incorporate mental health data from multiple informants, also including non-biological parental figures that act as primary caregivers.

## 5 Conclusion

We analyzed the associations between parental internalizing, externalizing, and total problems and functional connectivity patterns of ER networks in 4202 9–10-year-old children. Between and within functional connectivity patterns of ER networks were associated with parental psychopathology, but did not mediate the relationship between parental psychopathology and later youth psychopathology. Our findings suggest that parental psychopathology is potentially related to biased emotion generation and disrupted integration of information between subcortical and cortical networks in youth, but more research is needed to determine how this relates to the child’s own mental health.

## CRediT authorship contribution statement

**Ole A. Andreassen:** Writing – review & editing, Funding acquisition, Conceptualization. **Irene Voldsbekk:** Writing – review & editing, Visualization, Conceptualization. **Niamh MacSweeney:** Writing – review & editing, Methodology, Data curation, Conceptualization. **Lia Ferschmann:** Writing – review & editing, Conceptualization. **Linn. B. Norbom:** Writing – review & editing, Methodology, Formal analysis. **Eira R. Aksnes:** Writing – review & editing, Conceptualization. **Thea Wiker:** Writing – review & editing, Visualization, Software, Methodology, Formal analysis. **Christian K. Tamnes:** Writing – review & editing, Writing – original draft, Supervision, Project administration, Methodology, Funding acquisition, Data curation, Conceptualization. **Carmen Morawetz:** Writing – review & editing, Resources, Data curation, Conceptualization. **Haakon Engen:** Writing – review & editing, Writing – original draft, Supervision, Methodology, Formal analysis, Data curation, Conceptualization. **Espen Eilertsen:** Writing – review & editing, Software, Methodology, Formal analysis, Conceptualization. **Dylan G. Gee:** Writing – review & editing, Writing – original draft, Supervision, Conceptualization. **Dani Beck:** Writing – review & editing, Visualization, Supervision, Software, Methodology, Conceptualization. **Lars T. Westlye:** Writing – review & editing, Funding acquisition, Conceptualization. **Valerie Karl:** Writing – review & editing, Writing – original draft, Visualization, Project administration, Methodology, Formal analysis, Data curation, Conceptualization.

## Declaration of Generative AI and AI-assisted technologies in the writing process

During the preparation of this work the author(s) used ChatGPT in order to improve readability for selected sentences. After using this tool/service, the author(s) reviewed and edited the content as needed and take(s) full responsibility for the content of the publication.

## Declaration of Competing Interest

The authors declare that they have no known competing financial interests or personal relationships that could have appeared to influence the work reported in this paper.

## Footnotes

Appendix A Supplementary data associated with this article can be found in the online version at doi:10.1016/j.dcn.2024.101476.
