# Supplementary material for: Associations between parental psychopathology and youth functional emotion regulation brain networks
Source: Dev Cogn Neurosci. 2024 Nov 12;70:101476. doi: 10.1016/j.dcn.2024.101476 (PMC11609324; doi:10.1016/j.dcn.2024.101476)
Supplement: Supplementary file 1 — Supplementary material [file mmc1.docx]

**Supplementary Material**

Associations between parental psychopathology and youth functional emotion regulation brain networks

Valerie Karl^1,2*^, Dani Beck^1,3^, Espen Eilertsen^1^, Carmen Morawetz^4^, Thea Wiker^1,3^, Eira R. Aksnes^1,3^, Linn. B. Norbom^1^, Lia Ferschmann^1^, Niamh MacSweeney^1,3^, Irene Voldsbekk^3,5,6^, Ole A. Andreassen^6,7^, Lars T. Westlye^5,6,7^, Dylan G. Gee^2^, Haakon Engen^5,8^, Christian K. Tamnes^1,3^

^1^ PROMENTA Research Center, Department of Psychology, University of Oslo, Oslo, Norway

^2^ Department of Psychology, Yale University, New Haven, United States

^3^ Division of Mental Health and Substance Abuse, Diakonhjemmet Hospital, Oslo, Norway

^4^ Department of Psychology, University of Innsbruck, Innsbruck, Austria

^5^ Department of Psychology, University of Oslo, Oslo, Norway

^6^ Center for Precision Psychiatry, Division of Mental Health and Addiction, University of Oslo & Oslo University Hospital, Oslo, Norway

^7^ KG Jebsen Centre for Neurodevelopmental Disorders, University of Oslo, Oslo, Norway

^8^ Institute of Military Psychiatry Norwegian Armed Forces Joint Medical Services, Oslo, Norway

* Corresponding author: Valerie Karl, e-mail: valerie.karl@psykologi.uio.no, postal address: Department of Psychology, University of Oslo, PO box 1094 Blindern, 0317 Oslo, Norway

1. **Table S1 Peak Coordinates**
2. **Methodological Approach to derive Parcels corresponding to ERNs**
3. **Figure S1 Overview of Exclusion Process**
4. **Figure S2 Harmonization with neuroComBat**
5. **Figure S3 Sample Comparisons**
6. **Figure S4 Differences between Cluster and Peak Versions**
7. **Table S2 Results Cluster Version: Associations between Parental Psychopathology and Youth FC patterns**
8. **Results Mediation Analysis**
   1. **Table S3 A Coefficients (Peak Version)**
   2. **Table S4 A Coefficients (Cluster Version)**
   3. **Table S5 B Within and B Between Coefficients (Peak Version)**
   4. **Table S6 B Within and B Between Coefficients (Cluster Version)**
9. **Model Comparisons**
10. **Figure S5 Connectivity per Psychopathology Risk Group**
11. **Table S7 Associations between Connectivity and Non-neural Emotion Regulation**
12. **Family History of Psychopathology**
13. **Table S1**

| *Emotion Regulation Network Regions and Their Peak Coordinates* | | | | | | |
| --- | --- | --- | --- | --- | --- | --- |
| Side | Region | BA | Volume | x | y | z |
| ERN 1 | | | | | | |
| L | Superior_Frontal_Gyrus_a | 8 | 11704 | 0 | 24 | 50 |
| R | Middle_Frontal_Gyrus_a | 8 | 11024 | 40 | 24 | 42 |
| R | Inferior_Parietal_Lobule | 40 | 9968 | 58 | -52 | 38 |
| L | Inferior_Parietal_Lobule | 40 | 6216 | -58 | -50 | 44 |
| L | Middle_Frontal_Gyrus_b | 10 | 4664 | -36 | 52 | -2 |
| L | Middle_Frontal_Gyrus_c | 6 | 4288 | -42 | 14 | 48 |
| R | Middle_Frontal_Gyrus_d | 11 | 2792 | 42 | 46 | -8 |
| R | Insula | 13 | 2000 | 36 | 16 | 6 |
| R | Cingulate_Gyrus | 23 | 1336 | 2 | -22 | 30 |
| R | Precuneus_a | 7 | 944 | 10 | -64 | 36 |
| ERN2 | | | | | | |
| L | Inferior_Frontal_Gyrus | 47 | 19464 | -46 | 24 | -8 |
| L | Superior_Frontal_Gyrus_b | 6 | 16592 | -4 | 10 | 62 |
|  | Inferior_Frontal_Gyrus | 47 | 6856 | 50 | 28 | -8 |
| L | Superior_Temporal_Gyrus | 39 | 6704 | -46 | -52 | 28 |
| L | Middle_Temporal_Gyrus | *NA* | 5024 | -54 | -34 | -2 |
| L | Middle_Frontal_Gyrus | 6 | 4568 | -44 | 6 | 50 |
| L | Superior_Frontal_Gyrus_c | 9 | 3080 | -30 | 48 | 26 |
| L | Caudate | *NA* | 1960 | -16 | 10 | 12 |
| R | Tuber | *NA* | 1640 | 36 | -60 | -30 |
| ERN3 | | | | | | |
| L | Amygdala | *NA* | 8640 | -22 | -4 | -16 |
| R | Amygdala | *NA* | 6512 | 24 | -4 | -18 |
| R | Fusiform_Gyrus | 37 | 4776 | 40 | -46 | -18 |
| R | Thalamus_a | *NA* | 3528 | 6 | -26 | 0 |
| L | Fusiform_Gyrus | 37 | 1256 | -38 | -54 | -14 |
| L | Parahippocampal_Gyrus | 27 | 1216 | -22 | -28 | -4 |
| B | Medial_Frontal_Gyrus_f | 10 | 1016 | 0 | 54 | -10 |
| L | Inferior_Occipital_Gyrus | 19 | 912 | -42 | -76 | -6 |
| ERN4 | | | | | | |
| L | Postcentral_Gyrus | 2 | 4160 | -58 | -22 | 32 |
| L | Insula | 13 | 3752 | -44 | -4 | 10 |
| L | Superior_Parietal_Lobule | 7 | 2240 | -28 | -52 | 56 |
| R | Postcentral_Gyrus | 2 | 1736 | 62 | -22 | 30 |
| L | Cuneus | 18 | 1224 | -10 | -76 | 22 |
| L | Middle_Occipital_Gyrus | 19 | 1152 | -48 | -74 | 2 |
| R | Thalamus_b | *NA* | 1024 | 10 | -26 | -4 |
| R | Precuneus_b | 19 | 832 | 28 | -60 | 38 |
| R | Posterior_Cingulate | 30 | 832 | 16 | -56 | 16 |
| *Notes.* MNI coordinates of regions of interest as presented in Morawetz et al. (2020).  L=Left; R=Right, B=Bilateral | | | | | | |

1. **Methodological Approach to derive Parcels corresponding to ERNs (adapted from the Preregistration)**

The key dependent variables of the first part of our study were intra- and inter-network connectivity within and between four different emotion regulation networks (Morawetz et al., 2020). To derive measures of network connectivity, we used preprocessed rs-fMRI data from the ABCD dataset (Feczko et al., 2021; Collection 3165 from the ABCD-BIDS Community Collection; <https://collection3165.readthedocs.io/en/stable/derivatives/>). More specifically, we used connectivity matrices of 10 mins of parcellated resting state data (FD threshold of 0.2mm). These matrices contained correlation coefficients between each timeseries of a region with all timeseries of other regions. Here, regions were all parcels of the Gordon 2014 parcellation, as well as 19 FreeSurfer subcortical regions (Fischl et al., 2002; Gordon et al., 2016). For clarity, we will refer to both the Gordon parcels and the subcortical regions as “parcels”.

The first step was to match the meta-analytic maps of the four previously identified emotion regulation networks (Morawetz et al., 2020) onto Gordon parcels (Gordon et al., 2016) and subcortical regions (Fischl et al., 2002). We conducted this step prior to preregistration.

In addition to mapping clusters derived from Morawetz et al.’s metanalytic grouping procedure onto the Gordon parcels, we additionally obtained connectivity measures using a ROI sphere approach. Based on peak voxels of each cluster reported in Morawetz et al. (2020), we used MarsBar (Brett et al., 2002) and constructed 6 mm spheres around the peak coordinate. Maps of the spheres were then mapped onto Gordon parcels and subcortical regions using the same approach as described below.

The maps for each emotion regulation network were in MNI space, while the connectivity matrices were constructed using parcellated resting state data in cifti format. Due to individual cortical folding variability, mapping the volume-based group-average emotion regulation network clusters onto cortical surfaces would not result in precise cortical maps of the networks (Coalson et al., 2018). We thus chose to use a voxel-to-parcel approach, mapping each voxel within an emotion regulation cluster onto a combined mask of both the Gordon parcels and subcortical regions.

We retrieved a nifti-version of the Gordon parcels in MNI space (<https://www.mir.wustl.edu/research/research-centers/neuroimaging/labs/egordon-lab/resources/>). To ensure that all files were in the same format, we flipped the Gordon parcellation mask along the x-axis. Next, we used the workbench command “cifti-separate” on the original dlabel file (ABCC’s Gordon2014FreeSurferSubcortical_dparc.dlabel) to obtain volume-spaced maps of subcortical areas.

Once all files were registered and in MNI volume-space, we used MATLAB to combine the Gordon parcels and subcortical regions into a new nifti image. If a voxel overlapped with both a Gordon parcel and subcortical region, we assigned the voxel to the subcortical region.

For each emotion regulation network, we detected all voxels that belonged to a network and mapped the voxels onto the parcellation (i.e., we created a mask matrix for each cluster and multiplied it with the matrix containing the corresponding parcels for each voxel). Finally, we created a table, listing all parcels that mapped onto each emotion regulation network and their corresponding number of voxels within that parcel.

Next, we read the tables into R. As many parcels only overlapped with a few voxels, we calculated the first quartile of the total amount of voxels that overlapped with parcels. We used this as a threshold and excluded all parcels within the first quartile (≤ 5 voxels). For any parcels that were originally assigned to two different emotion regulation networks, we assigned the parcel to the emotion regulation network with which it overlapped more. In one case, 8 voxels of the same parcel (parcel 27) overlapped with edges of clusters of two different emotion regulation networks, so we decided to exclude that parcel from the analysis. This resulted in a list of parcels for each emotion regulation network.

For our main analysis, we calculated the mean of all parcel-pairings within a network, and the mean of all parcel-pairings between two networks (see Zhang et al. (2019) for a similar approach). This yielded four intra-network and six inter-network connectivity measures, i.e., our dependent variables.

1. **Figure S1**

*Overview of the Exclusion Process*


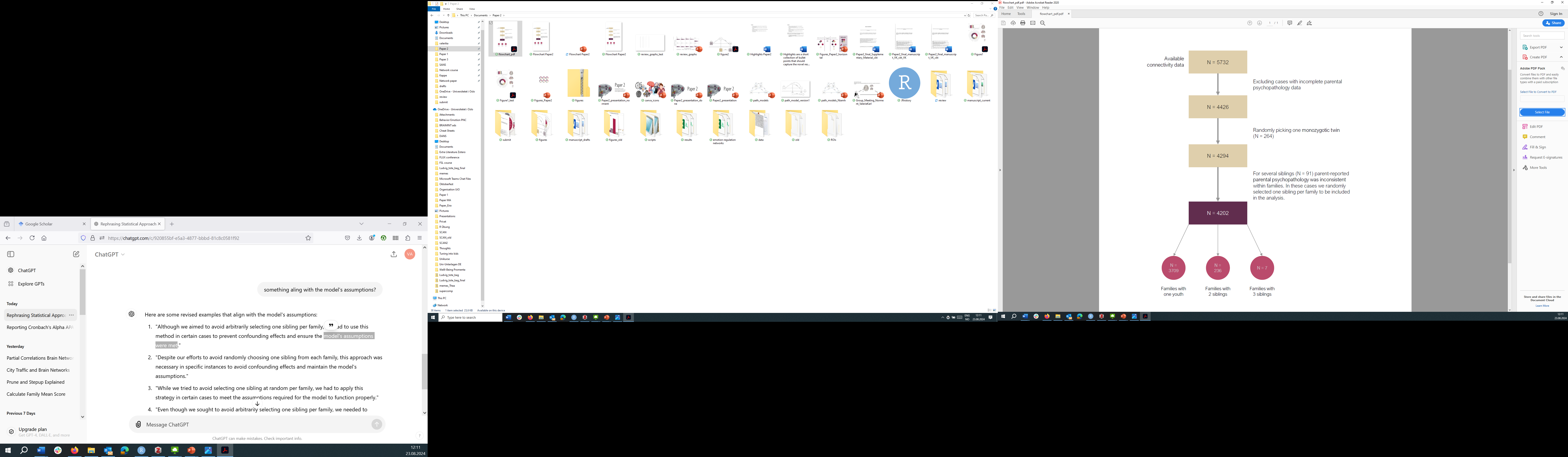


*Note.* Connectivity data was available for 5732 participants. 4426 participants additionally had complete parental psychopathology data. We randomly picked one monozygotic twin if both twins participated in the study (N= 264). Additionally, in cases where reported parental psychopathology was inconsistent within families (N=91), we also randomly included only one sibling per family. This led to a final sample size of 4202.

1. **Figure S2**

*Within ERN1 Connectivity before and after Harmonization with neuroComBat*


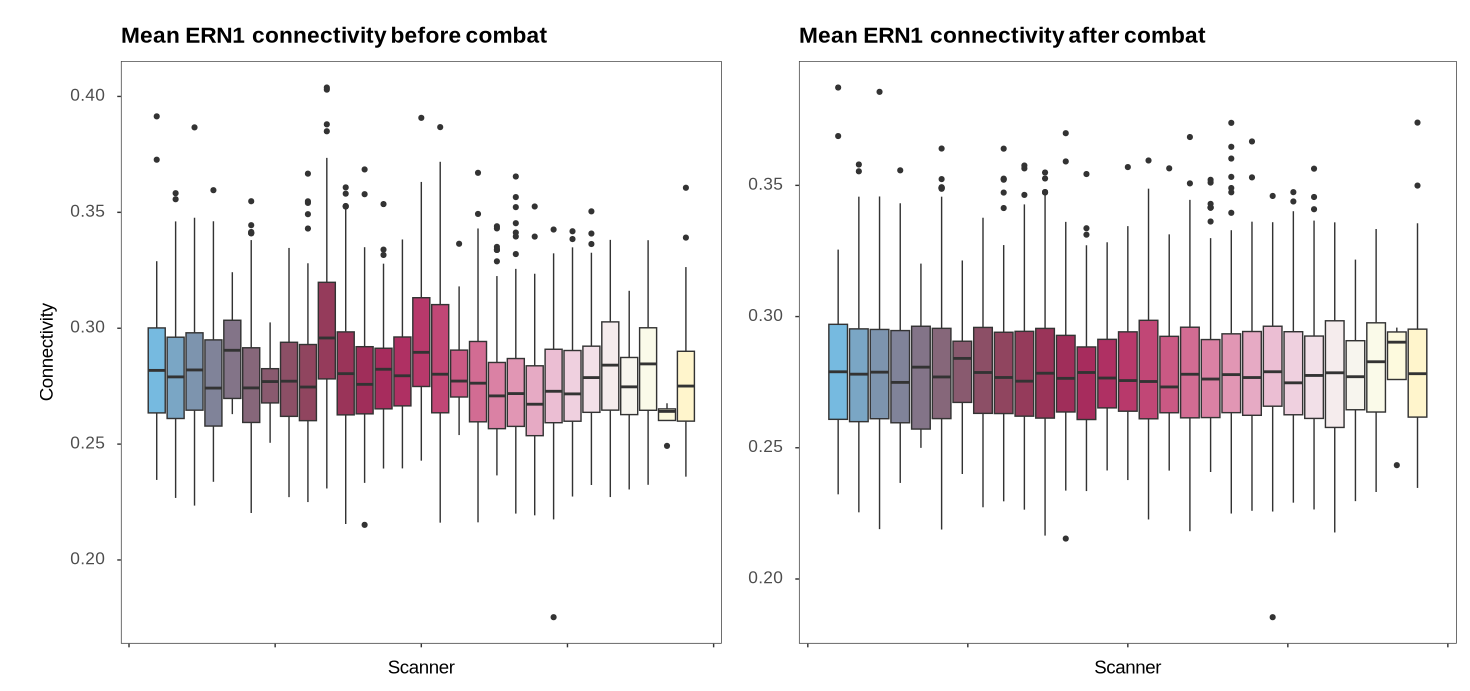


*Note.* Pre and post harmonized mean connectivity within ERN1 per scanner. Data was adjusted for scanner effects using neuroComBat (Fortin, 2021) using youth age, youth sex, and both parental and youth internalizing, externalizing, and total problems as covariates.

1. **Figure S3**

*Sample Comparison of Youth Psychopathology*


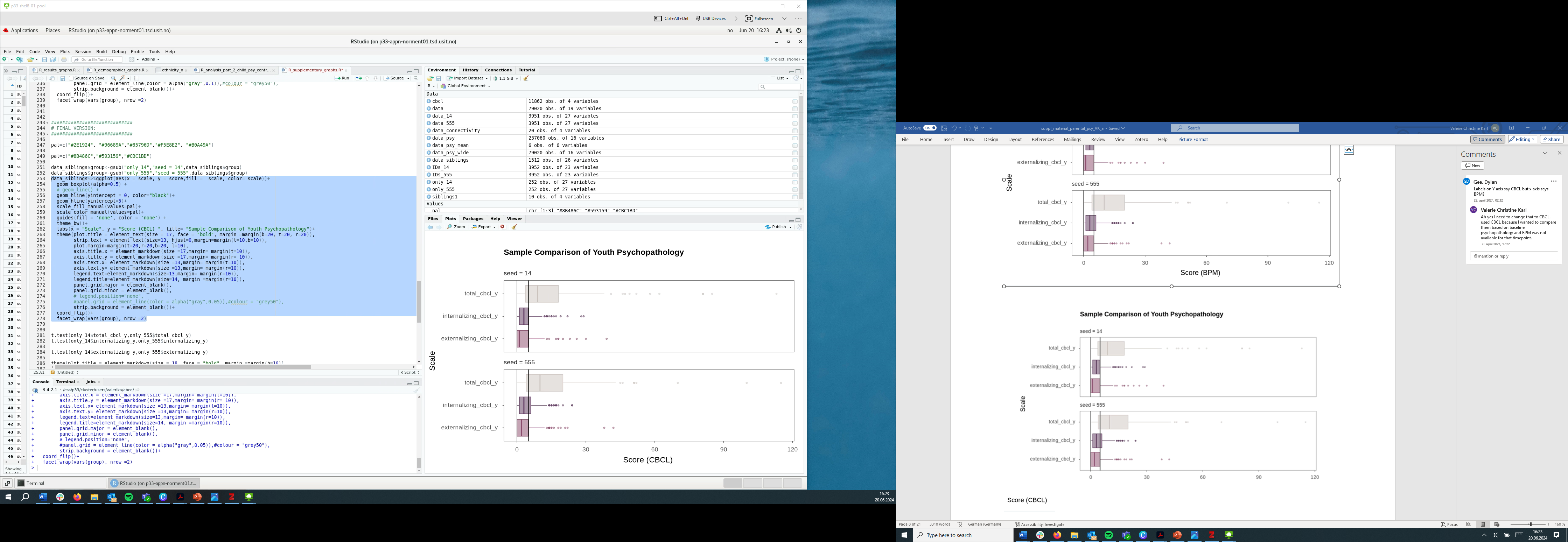


*Note.* Sample comparison between participants that were different depending on sampling seed (14 or 555). We initially randomly selected one sibling per family to include in our analyses. This led to different results depending on the seed that was set for the randomization. Exploratory plots indicate that psychopathology between randomly chosen siblings might differ slightly, although not significantly (*p* > .05). We used parent-reported internalizing, externalizing, and total problem scales from the Child Behavior Checklist (CBCL; Achenbach, 2009), as Brief Problem Monitor scores were not available for the baseline timepoint.

1. **Figure S4**


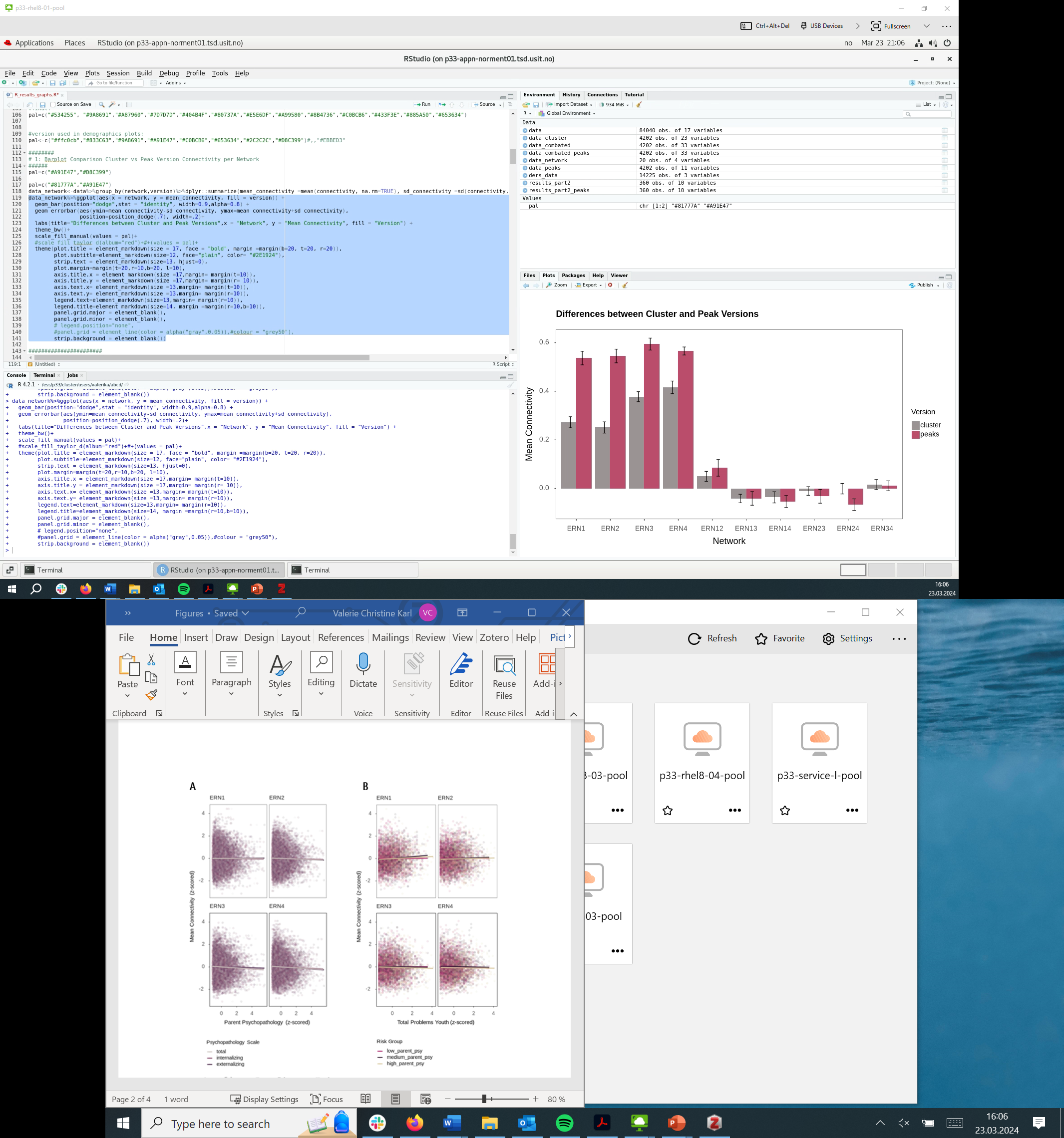


Note. Mean connectivity differences between cluster and peak approaches. Error bars indicate +/- 1 standard deviation.

1. **Table S2 Results Cluster Version: Associations between Parental Psychopathology and Youth FC patterns**

**Table S2**

| *Main Effect of Parental Psychopathology on Emotion Regulation Network Connectivity (Cluster Version)* | | | | | | |
| --- | --- | --- | --- | --- | --- | --- |
| Network | Estimate | Standard Error | *df* | *T* - value | *p_bootstrapped_* | *p*_FDR-corrected_ |
| Internalizing problems | | | | | | |
| ERN1 | 0.01 | 0.02 | 3928.75 | 0.61 | 0.568 | 0.774 |
| ERN2 | 0.00 | 0.02 | 3953.03 | -0.23 | 0.835 | 0.864 |
| ERN3 | -0.03 | 0.02 | 3935.61 | -1.64 | 0.108 | 0.258 |
| ERN4 | -0.03 | 0.02 | 3936.68 | -1.74 | 0.096 | 0.258 |
| ERN12 | 0.01 | 0.02 | 3994.54 | 0.33 | 0.750 | 0.819 |
| ERN13 | 0.03 | 0.02 | 3929.27 | 2.00 | 0.042 | 0.253 |
| ERN14 | -0.01 | 0.02 | 3951.23 | -0.31 | 0.764 | 0.819 |
| ERN23 | 0.03 | 0.02 | 3980.32 | 1.94 | 0.059 | 0.253 |
| ERN24 | 0.02 | 0.02 | 3971.25 | 1.47 | 0.129 | 0.258 |
| ERN34 | -0.03 | 0.02 | 3964.74 | -1.90 | 0.056 | 0.263 |
| Externalizing problems | | | | | | |
| ERN1 | 0.02 | 0.02 | 3827.76 | 1.51 | 0.149 | 0.279 |
| ERN2 | -0.01 | 0.02 | 3846.47 | -0.44 | 0.687 | 0.819 |
| ERN3 | -0.01 | 0.02 | 3834.25 | -0.82 | 0.423 | 0.634 |
| ERN4 | 0.00 | 0.02 | 3833.31 | 0.03 | 0.985 | 0.985 |
| ERN12 | 0.01 | 0.02 | 3902.57 | 0.80 | 0.451 | 0.644 |
| ERN13 | 0.03 | 0.02 | 3818.66 | 1.69 | 0.100 | 0.258 |
| ERN14 | -0.02 | 0.02 | 3860.44 | -1.48 | 0.127 | 0.258 |
| ERN23 | 0.04 | 0.02 | 3890.11 | 2.55 | 0.006 | 0.180 |
| ERN24 | 0.01 | 0.02 | 3888.33 | 0.55 | 0.593 | 0.774 |
| ERN34 | -0.03 | 0.02 | 3890.17 | -1.88 | 0.057 | 0.253 |
| Total problems | | | | | | |
| ERN1 | 0.02 | 0.02 | 3898.95 | 1.30 | 0.184 | 0.324 |
| ERN2 | -0.01 | 0.02 | 3921.23 | -0.36 | 0.715 | 0.819 |
| ERN3 | -0.03 | 0.02 | 3904.51 | -1.75 | 0.089 | 0.258 |
| ERN4 | -0.02 | 0.02 | 3905.75 | -1.56 | 0.124 | 0.258 |
| ERN12 | 0.01 | 0.02 | 3967.09 | 0.44 | 0.666 | 0.819 |
| ERN13 | 0.03 | 0.02 | 3896.08 | 1.73 | 0.076 | 0.258 |
| ERN14 | -0.01 | 0.02 | 3924.43 | -0.84 | 0.406 | 0.634 |
| ERN23 | 0.04 | 0.02 | 3954.05 | 2.35 | 0.017 | 0.253 |
| ERN24 | 0.01 | 0.02 | 3947.08 | 0.90 | 0.363 | 0.604 |
| ERN34 | -0.03 | 0.02 | 3942.98 | -1.98 | 0.038 | 0.253 |
| *Note.* Main effects of psychopathology on within- and between emotion regulation network connectivity using the cluster approach. We used linear mixed models to test the associations between parental internalizing, externalizing, and total problems and connectivity within and between four emotion regulation networks, while controlling for age and sex. Family ID served as a random effect to account for the nested nature of the data. Bootstrap resampling with 1000 iterations resulted in adjusted *p*-values, which were further corrected for multiple comparisons using a False-Discovery Rate (FDR) approach (Benjamini & Hochberg, 1995). In the cluster version associations fell short of significance after correcting for multiple comparisons. Before correcting for multiple comparisons, we found associations between inter ERN13 and parental internalizing problems, inter 23 and parental externalizing problems, and inter ERN23 and ERN34 and parental total problems.  ERN = emotion regulation network  * *p* < .05 | | | | | | |

1. **Results Mediation Analysis**

Due to the nested nature of our data, we estimated two different estimates linking emotion regulation network (ERN) connectivity and youth psychopathology: b_within representing the within family effect and b_between, which stands for a between family effect. Therefore, we were able to test two different indirect paths, i.e. whether ERN connectivity mediated the relationship between parental and youth psychopathology via a between family or potentially a within family effect. Given the link between parental psychopathology and ERN connectivity (*a*) and two b coefficients (*b_between, b_within*), we could test two indirect effects (*a*b_between*, *a*b*within*) and therefore also two total effects (*total1* = *a***b_between* + *c*; *total2*=  *a***b_within* + *c*).

For both approaches, we found significant total effects (Cluster approach: *B_total1_* = [0.11 - 0.20], *p* < .001, *B_total2_* = [0.12 - 0.18], *p* < .001; Peak-approach: *B_total1_* = [0.12 - 0.22], *p* < .001, *B_total2_* = [0.12 - 0.18], *p* < .001), yet the indirect paths (*a*b_between*, *a*b*within)* were not significant. Therefore, ERN connectivity did not mediate the relationship between parental and youth psychopathology.

When using the peak version (Table S5) and looking at uncorrected within-family effects, all three parental psychopathology dimensions were positively associated with between ERN1 and ERN4 connectivity. Between ERN1 and ERN3 connectivity was negatively associated with youth externalizing and total problems, with the latter also being negatively related to within ERN4 connectivity. On the other hand, between family effects showed positive associations between youth internalizing and total problems and within ERN4 connectivity, negative associations between ERN1 and ERN4 connectivity and externalizing and total problems, and lastly a negative relationship between total problems and between ERN2 and ERN4 connectivity.

Due to the opposite patterns of relationships between and within family effects, we conducted additional analyses to explore if there were significant differences among within and between family effects. We did not find significant differences between the two effects, after correcting for multiple comparisons. Uncorrected results indicating differences between model fits can be found in the Supplementary Material (section 7).

- 1. **A Coefficients (Peak Version)**

**Table S3**

| *Beta Estimates Linking Parental Psychopathology and Emotion Regulation Network Connectivity in the Multilevel Mediation Model (Peak Version)* | | | | | |
| --- | --- | --- | --- | --- | --- |
| Network | *β* estimate | *se* | *z-value* | *p_uncorrected_* | *p*_FDR-corrected_ |
| Internalizing | | | | | |
| ERN12 | -0.04 | 0.02 | -2.23 | .025 | .140 |
| Externalizing | | | | | |
| ERN4 | -0.04 | 0.02 | -2.74 | .006 | .129 |
| ERN12 | -0.03 | 0.02 | -2.11 | .034 | .140 |
| ERN23 | 0.03 | 0.02 | 2.08 | .037 | .140 |
| ERN24 | 0.02 | 0.02 | 2.13 | .033 | .140 |
| Total Problems | | | | | |
| ERN4 | -0.03 | 0.02 | -2.15 | .032 | .140 |
| ERN12 | -0.04 | 0.02 | -2.63 | .009 | .129 |
| ERN23 | 0.03 | 0.02 | 2.02 | .044 | .146 |
| ERN24 | 0.03 | 0.02 | 2.16 | .031 | .140 |
| *Note.* Estimates resulted from a multilevel mediation model testing whether network connectivity (peak-based) mediated the relationship between parental and youth psychopathology (i.e. the *a* coefficient), while correcting for age and sex effects. Presented are standardized coefficients (*p_uncorrected_* < .05) linking parentally psychopathology to emotion regulation network connectivity patterns. Results are FDR-corrected (Benjamini & Hochberg, 1995). None of the results remained significant after correcting for multiple comparisons. Before correcting for multiple comparisons, both parental externalizing and total problems were positively associated with between ERN2 and ERN3, as well as between ERN 2 and ERN4 connectivity, and negatively associated with within ERN4 connectivity. All psychopathology dimensions were negatively association with between ERN1 and ERN2 connectivity.  ERN = Emotion regulation network; *se* = Standard error; Alpha = 0.05 | | | | | |

- 1. **A Coefficients (Cluster Version)**

**Table S4**

| *Beta Estimates Linking Parental Psychopathology and Emotion Regulation Network Connectivity in the Multilevel Mediation Model (Cluster Version)* | | | | | |
| --- | --- | --- | --- | --- | --- |
| Network | *β* estimate | *SE* | *z-value* | *p_uncorrected_* | *p*_FDR-corrected_ |
| Internalizing | | | | | |
| ERN4 | -0.04 | 0.02 | -2.30 | .021 | .267 |
| Externalizing | | | | | |
| ERN1 | 0.03 | 0.02 | 2.10 | .035 | .267 |
| Total Problems | | | | | |
| ERN1 | 0.04 | 0.02 | 2.24 | .025 | .267 |
| ERN4 | -0.03 | 0.02 | -2.10 | .036 | .267 |
| *Note.* Estimates resulted from a multilevel mediation model testing whether network connectivity (cluster-based) mediated the relationship between parental and youth psychopathology (i.e. the *a* coefficient), while correcting for age and sex effects. Presented are standardized coefficients (*p_uncorrected_* < .05) linking parentally psychopathology to emotion regulation network connectivity patterns. Results are FDR-corrected (Benjamini & Hochberg, 1995). We found a significant negative relationship between parental internalizing and total problems and intra ERN4 connectivity, and a positive association between parental externalizing and total problems and intra ERN 1 connectivity.  ERN = Emotion regulation network; *SE* = Standard error; Alpha = 0.05 | | | | | |

- 1. **Between and Within Family Effects (Peak Version)**

**Table S5**

| *Between and Within Family Effect Estimates (Peak version)* | | | | | | |
| --- | --- | --- | --- | --- | --- | --- |
| Network | Effect | *β* estimate | *SE* | *z*-value | *p* | *p*_FDR-corrected_ |
| Internalizing | | | | | | |
| ERN14 | b_within | 0.10 | 0.05 | 2.22 | .026 | .198 |
| ERN4 | b_between | 1.35 | 0.68 | 2.00 | .046 | .279 |
| Externalizing | | | | | | |
| ERN13 | b_within | -0.09 | 0.05 | -2.03 | .042 | .222 |
| ERN14 | b_within | 0.11 | 0.05 | 2.36 | .018 | .198 |
| ERN14 | b_between | -1.47 | 0.68 | -2.17 | .030 | .279 |
| Total | | | | | | |
| ERN4 | b_within | -0.10 | 0.04 | -2.28 | .023 | .198 |
| ERN13 | b_within | -0.09 | 0.04 | -2.01 | .044 | .222 |
| ERN14 | b_within | 0.12 | 0.04 | 2.79 | .005 | .159 |
| ERN4 | b_between | 1.34 | 0.68 | 1.99 | .047 | .279 |
| ERN14 | b_between | -1.48 | 0.67 | -2.21 | .027 | .155 |
| ERN24 | b_between | -0.53 | 0.26 | -2.03 | .042 | .155 |
| *Note*. Beta estimates resulting from a multilevel mediation model testing whether network connectivity (peak version) mediated the relationship between parental and youth psychopathology. Estimates are beta coefficients (*p_uncorrected_* < .05) of within- and between family effect linking emotion regulation network connectivity patterns to youth psychopathology. Age and sex were covariates of no interest. We used FDR to correct for multiple comparisons.  ERN = Emotion regulation network; *SE* = Standard error; Alpha = 0.05 | | | | | | |

- 1. **Between and Within Family Effects (Cluster Version)**

**Table S6**

| *Between and Within Family Effect Estimates (Cluster version)* | | | | | | |
| --- | --- | --- | --- | --- | --- | --- |
| Network | Effect | *β* estimate | *SE* | *z-value* | *p_uncorrected_* | *p*_FDR-corrected_ |
| Internalizing | | | | | | |
| ERN4 | b_between | 0.66 | 0.31 | 2.16 | .031 | .195 |
| ERN14 | b_between | -0.57 | 0.23 | -2.47 | .014 | .195 |
| ERN34 | b_between | 0.37 | 0.17 | 2.14 | .032 | .195 |
| Total | | | | | | |
| ERN4 | b_within | -0.10 | 0.05 | -2.06 | .039 | .307 |
| ERN14 | b_within | 0.10 | 0.05 | 2.14 | .032 | .307 |
| ERN4 | b_between | 0.68 | 0.30 | 2.31 | .021 | .195 |
| ERN14 | b_between | -0.61 | 0.23 | -2.71 | .007 | .195 |
| *Note.* Estimates are derived from a multilevel mediation model testing whether network connectivity (cluster-based) mediated the relationship between parental and youth psychopathology, while correcting for age and sex effects. Presented are within- and between family effect coefficients (*p_uncorrected_* < .05) linking emotion regulation network connectivity patterns to youth psychopathology. Results are FDR-corrected (Benjamini & Hochberg, 1995). None of the relationships remained significant after correcting for multiple comparisons. In short, when looking at uncorrected within-family effects and using the cluster-approach, youth total problems correlated negatively with within ERN4 connectivity and positively with between ERN1 and ERN4 connectivity. Between-family effects showed that child internalizing and total problems were positively associated with within ERN4 and negatively with between ERN1 and ERN4 connectivity. Internalizing problems were additionally positively correlated with between ERN3 and ERN4 connectivity.  ERN = Emotion regulation network; *SE* = Standard error; Alpha = 0.05 | | | | | | |

1. **Model Fit Comparisons**

To statistically test the difference between within and between family effects, we set up two different models (one of them distinguishing between the two effects and resulting two indirect and total effects) and used a Chi-Squared Difference Test to assess whether they led to different model fits. After FDR-correction (Benjamini & Hochberg, 1995) there was no significant difference between any of the models. Before correcting for multiple comparisons, we found a difference between the models for intra-ERN4 (Δχ²(5.58), *p* = .018) and inter ERN14 (Δχ²(4.12), *p* = .042) connectivity for internalizing problems, inter ERN14 (Δχ²(6.93), *p* = .008) and ERN34 (Δχ²(4.26), *p* = .039) connectivity for externalizing problems and intra ERN4 (Δχ²(5.83), *p* = .016), and inter ERN14 (Δχ²(7.64), *p* = .006), and ERN24 (Δχ²(4.37), *p* = .037) for total problems.

1. **Figure S5**

*Within and Between Connectivity of Emotion Regulation Network per Psychopathology Risk Group*


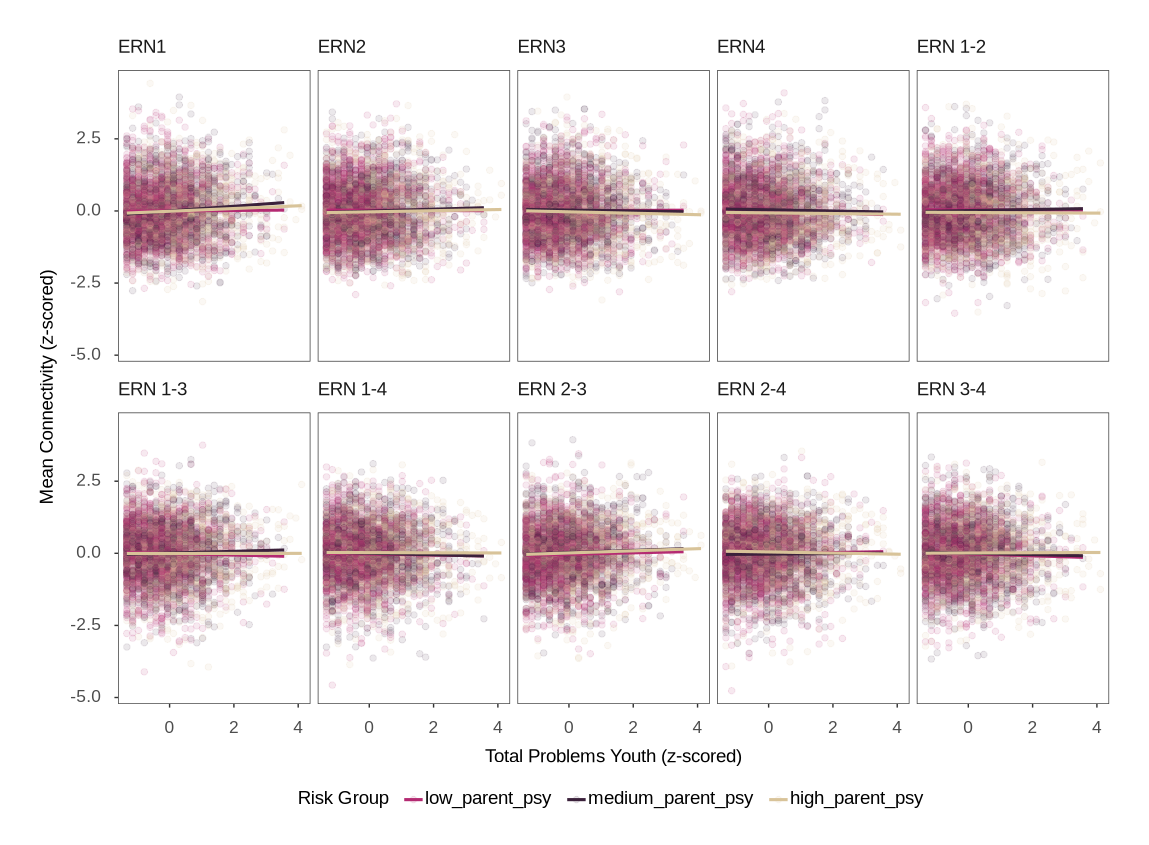


*Note.* Associations between mean FC within and between each ER network per risk group based on youth’s exposure to parental total problems. Data was split into terciles based on parental total problem scores to serve as a proxy for youth’s risk of exposure to parental psychopathology.

1. Associations between Emotion Regulation Network Connectivity and Emotion Regulation skills

For exploratory purposes, we additionally conducted analyses testing the association between youth’s ERN connectivity patterns at baseline and youth’s self-reported ER skills at the 3-year follow-up timepoint. To this end, we utilized the reappraisal and suppression scores of the Emotion Regulation Questionnaire (Garnefski et al., 2007; Gross & John, 2003; Gullone & Taffe, 2012). These scores each represented the sum of 3 items. The items were rated on a 5-point-Likert scale and reflected engagement in adaptive (reframing situations, i.e. reappraisal) and maladaptive (inhibiting emotion expressions, i.e. suppression) ER strategies.

We used linear mixed models and treated ER skills as the dependent variable and ERN connectivity, age, and sex at baseline as fixed effects and family ID as a random effect. We ran the analyses for each ERN connectivity measure (4 within-connectivity measures, 6 between-connectivity measures) and the two non-neural ER measures (resulting in a total of 20 tests). The results were subsequently adjusted for multiple comparisons using FDR.

We did not find any significant associations between youth’s ERN connectivity at baseline and their ER skills at a 3 year-follow up. Future studies should investigate the relationship between neural ERN connectivity and ER skills using potentially more nuanced measures of ER. It is important to consider the distinctions between youth’s tendency and capacity to use ER skills (Caballero et al., 2023; Silvers, 2022; Silvers & Guassi Moreira, 2019). The phrasing of the ERQ items would target the tendency of youth to use the ER skills (e.g. “When I want to feel happy about something, I change the way I’m thinking about it”), not necessarily whether they are capable of using the skill.

**Table S7**

| *Associations between Youth’s Emotion Regulation Network Connectivity at Baseline and Emotion Regulation skills at a 3-year Follow-up Timepoint* | | | | | |
| --- | --- | --- | --- | --- | --- |
| Network | *β* estimate | *se* | *z-value* | *p_uncorrected_* | *p*_FDR-corrected_ |
| Reappraisal | | | | | |
| ERN1 | 0.02 | 0.02 | 1.07 | 0.284 | 0.796 |
| ERN2 | -0.01 | 0.02 | -0.47 | 0.637 | 0.796 |
| ERN3 | -0.00 | 0.02 | -0.07 | 0.944 | 0.944 |
| ERN4 | -0.01 | 0.02 | -0.65 | 0.518 | 0.796 |
| ERN12 | -0.01 | 0.02 | -0.57 | 0.567 | 0.796 |
| ERN13 | 0.01 | 0.02 | 0.61 | 0.539 | 0.796 |
| ERN14 | -0.00 | 0.02 | -0.14 | 0.889 | 0.944 |
| ERN23 | -0.01 | 0.02 | -0.83 | 0.406 | 0.796 |
| ERN24 | 0.01 | 0.02 | 0.81 | 0.416 | 0.796 |
| ERN34 | -0.01 | 0.02 | -0.87 | 0.385 | 0.796 |
| Suppression |  |  |  |  |  |
| ERN1 | 0.01 | 0.02 | 0.58 | 0.560 | 0.706 |
| ERN2 | -0.01 | 0.02 | -0.34 | 0.737 | 0.737 |
| ERN3 | -0.01 | 0.02 | -0.39 | 0.694 | 0.737 |
| ERN4 | -0.02 | 0.02 | -1.25 | 0.211 | 0.524 |
| ERN12 | -0.01 | 0.02 | -0.63 | 0.528 | 0.706 |
| ERN13 | -0.01 | 0.02 | -0.58 | 0.565 | 0.706 |
| ERN14 | 0.02 | 0.02 | 1.38 | 0.166 | 0.524 |
| ERN23 | 0.03 | 0.02 | 2.00 | 0.045 | 0.452 |
| ERN24 | 0.02 | 0.02 | 1.32 | 0.187 | 0.524 |
| ERN34 | -0.02 | 0.02 | -1.12 | 0.262 | 0.524 |
| *Note.* Standardized Coefficients representing the (non-significant) main effect of youth’s emotion regulation network connectivity at baseline on emotion regulation at a 3-year follow-up time-point. Emotion regulation was assessed with the Emotion Regulation Questionnaire (self-report) and yielded a reappraisal and suppression scale. We ran linear mixed model with each emotion regulation scale as the dependent variable and network connectivity, age, sex as fixed effects and family ID as a random effect. Results were adjusted for multiple comparisons using FDR-correction.  ERN = Emotion regulation network; *se* = Standard error; Alpha = 0.05 | | | | | |

1. Family History of Psychopathology

In our main analyses we focused on parental psychopathology within the last 6 months. We additionally conducted follow-up analyses that included any known parental psychiatric history and the interaction with parental psychopathology within the last 6 months, as covariates and repeated our analyses.

During the baseline assessment, the caregiver accompanying the participant answered several questions addressing the family history of psychopathology (Brown et al., 2015; Rice et al., 1995). The caregiver was first asked whether they had any knowledge about the child’s biological relatives. If they answered yes, the caregiver was then asked about an existing history of psychopathology within the family. First, they were generally asked if any blood relative has experienced different aspects of psychiatric history (“Has any blood relative of your child ever…”) and could choose between “Yes”, “No”, “Don’t know”, “Refuse to answer”. If they answered yes to the item, questions assessing which type of biological relative (e.g. biological father, maternal grandmother, paternal aunt) was affected, followed up. To align with our main analysis, we selected the response from each biological parent whose self-reported current psychopathology was included in the analyses. If a parent answered “Don’t know” to the initial question if “any blood relative has ever…”, the follow-up items would automatically be coded as missing. Given that a biological parent would know if they themselves had experience with e.g. mania or depression (and would therefore answer “yes”), we treated these cases of missing as indicators that they themselves had no experience with the symptom and no knowledge about other blood relatives’ history with e.g. mania or depression. The final sample included 4139 parents with complete data.

We decided to focus on the responses to the items assessing experiences with symptoms of depression, mania, psychosis, trouble, and nerves (see Table S8). If the response was 1 (“Yes”), this indicated that the biological parent has experienced the described symptoms, and 0 indicated no previous experience. Based on the sum of responses to the items, we created a new binary variable that reflected if the parent had any known psychiatric history (sum > 0) or no history of psychopathology (sum = 0). We used this grouping variable in our follow-up analyses as a covariate and tested both the main effect and interaction effect with current parental psychopathology on youth ERN connectivity and youth psychopathology.

**Linear mixed models**

First, we re-ran the linear mixed models with network connectivity measures as dependent variables and current psychopathology, psychiatric history, and their interactions term as fixed effects. Additionally, we included family ID as a random effects and youth’s age and sex as covariates. Following a bootstrapping procedure with 1000 iterations, we corrected the resulting bootstrapped p values for multiple comparisons using FDR (Benjamini & Hochberg, 1995) and a threshold of α = 0.05.

**Multilevel Mediation Analyses**

Furthermore, we repeated the multilevel mediation analyses and added parental psychiatric history and the interaction term of parent’s current psychopathology and psychiatric history to the model predicting youth’s ERN connectivity at baseline and youth’s psychopathology at a 3-year follow-up. The results were adjusted for multiple comparisons using FDR-correction.

**Table S8**

*Overview of Items Assessing Family History of Psychopathology*

| Psychopathology | Item |
| --- | --- |
| Depression | “Has ANY blood relative of your child ever suffered from depression, that is, have they felt so low for a period of at least two weeks that they hardly ate or slept or couldn't work or do whatever they usually do?” |
| Mania | “Has ANY blood relative of your child ever had a period of time when others were concerned because they suddenly became more active day and night and seemed not to need any sleep and talked much more than usual for them?” |
| Psychosis | “Has ANY blood relative of your child ever had a period lasting six months when they saw visions or heard voices or thought people were spying on them or plotting against them?” |
| Trouble | “Has ANY blood relative of your child been the kind of person who never holds a job for long, or gets into fights, or gets into trouble with the police from time to time, or had any trouble with the law as a child or an adult?” |
| Nerves | “Has ANY blood relative of your child ever had any other problems with their nerves, or had a nervous breakdown?” |
| *Note.* Items assessing family psychopathology history. Follow-up questions indicated which biological relative experienced the symptoms (e.g. biological father, maternal grandmother, paternal aunt) | |

**Results**

**Linear mixed models**

Neither current parental psychopathology, psychiatric history, nor their interaction term were significantly associated with any connectivity measures.

**Table S9**

| *Main Effect of Current Parental Psychopathology and Parent’s Psychiatric History, and Their Interaction Effect on Youth Emotion Regulation Network Connectivity (Peak Version)* | | | | | | | | |
| --- | --- | --- | --- | --- | --- | --- | --- | --- |
|  | Psychopathology Scale | Network | *β* estimate | Standard Error | *df* | *T* - value | *p_bootstrapped_* | *p*_FDR-corrected_ |
| **Current parental psychopathology** | | | | | | | | |
|  | Internalizing | ERN12 | -0.05 | 0.02 | 3845.43 | -2.27 | 0.031 | 0.310 |
|  | Externalizing | ERN12 | -0.06 | 0.02 | 3638.88 | -2.72 | 0.005 | 0.075 |
|  | Total | ERN12 | -0.07 | 0.02 | 3752.44 | -2.94 | 0.003 | 0.075 |
| **Parent psychopathology history** | | | | | | | | |
|  | Internalizing | ERN2 | -0.08 | 0.04 | 3741.55 | -2.01 | 0.044 | 0.048 |
|  | Externalizing | ERN24 | 0.08 | 0.04 | 3818.75 | 2.07 | 0.039 | 0.054 |
| **Interaction Term** | | | | | | | | |
|  | Externalizing | ERN3 | -0.10 | 0.03 | 3878.49 | -2.88 | 0.005 | 0.150 |
|  | Total | ERN3 | -0.08 | 0.03 | 3888.69 | -2.26 | 0.026 | 0.390 |
| *Note.* Main effects of psychopathology on within- and between emotion regulation network connectivity in youth. We employed linear mixed models to test the associations between parental internalizing, externalizing, and total problems and youth ERN connectivity (peak-based) within and between four emotion regulation networks. Youth age and sex were covariates and family ID served as a random effect to account for the nested nature of the data. We used a bootstrapping procedure with 1000 iterations to obtain *p*-values and corrected the results using FDR correction. Presented are standardized coefficients (*p_uncorrected_* < .05).  ERN = emotion regulation network. *se* = Standard error; Alpha = 0.05 | | | | | | | | |

**Multilevel Mediation Analyses**

Aligning with our main analyses, ERN did not mediate the relationship between parental psychopathology and youth psychopathology after adjusting for parents’ psychiatric history, yet the total effect remained significant (*β_total1_* = [0.12 - 0.23], *p* < .001, *β_total2_* = [0.12 - 0.19], *p* < .001). Moreover, parent’s current psychopathology (*β*_c_ = [0.12 - 0.23], p < .001 for all network connectivity measures) and psychiatric history (see Table S10) were directly associated with youth psychopathology (yet not their interaction term). Additionally, the association between current parental psychopathology and youth’s ERN connectivity disappeared when adding parent’s psychiatric history to the model.

**Conclusion**

Overall, the results suggest that parental current psychopathology and psychiatric history contain important information that might explain a higher risk of psychopathology of youth of parents with psychopathology. However, these results should be considered with caution. Singularity warnings and indications of small cluster sizes (most families only contained one sibling) indicate that the data used in this approach were not sufficient to determine the role of parent’s psychiatric history when looking at associations between current parental psychopathology and youth’s ERN connectivity and psychopathology.

Moreover, the measure for psychiatric history we used is based on “yes” or “no” answers on 5 different items. Thus, the measure does not contain information on the timing and severity of the experience with psychiatric symptoms. The phrasing of the questions also do not make a distinction between current and previously experienced mental health problems. Subsequently, the measure contained information on both current and past psychopathology and it was not possible to properly distinguish between current and past risk factors with the available data.

Concurrently, including both variables and their interaction term into the model, seems to decrease the (already small) associations between current psychopathology and youth’s ERN connectivity, leading to the lack of significant associations in this follow-up analysis. A potential interpretation is that by adding parent’s psychiatric history, we are partially accounting for the confounding genetic effect of psychiatric disorders that is transmitted across generations. This might have caused the effect of current psychopathology to decrease, as the genetic vulnerability is removed from the variable.

Future research should explore these facets of familial psychopathology risk more thoroughly with more appropriate measures that distinguish between past and current psychopathology, and designs that permit genetic and environmental factors to be resolved.

**Table S10**

| *Beta Estimates Linking Parental Psychopathology History and Youth Psychopathology at a 3-year Follow Up in the Multilevel Mediation Model (Peak Version)* | | | | | |
| --- | --- | --- | --- | --- | --- |
| Network | *β* estimate | *se* | *z-value* | *p_uncorrected_* | *p*_FDR-corrected_ |
| Internalizing | | | | | |
| ERN1 | 0.19 | 0.06 | 3.13 | 0.002 | 0.005** |
| ERN2 | 0.19 | 0.07 | 2.75 | 0.006 | 0.011** |
| ERN3 | 0.20 | 0.07 | 2.89 | 0.004 | 0.008** |
| ERN12 | 0.19 | 0.06 | 3.13 | 0.002 | 0.005** |
| ERN13 | 0.18 | 0.06 | 2.88 | 0.004 | 0.008** |
| ERN23 | 0.19 | 0.06 | 3.13 | 0.002 | 0.005** |
| ERN24 | 0.20 | 0.07 | 2.93 | 0.003 | 0.008** |
| ERN34 | 0.19 | 0.06 | 3.09 | 0.002 | 0.005** |
| Externalizing | | | | | |
| ERN1 | 0.12 | 0.06 | 2.20 | 0.028 | 0.044* |
| ERN3 | 0.19 | 0.07 | 2.72 | 0.007 | 0.012* |
| ERN24 | 0.15 | 0.06 | 2.29 | 0.022 | 0.037* |
| Total Problems | | | | | |
| ERN1 | 0.23 | 0.06 | 3.65 | 0.000 | 0.003** |
| ERN2 | 0.24 | 0.07 | 3.34 | 0.001 | 0.004** |
| ERN3 | 0.24 | 0.07 | 3.30 | 0.001 | 0.004** |
| ERN12 | 0.24 | 0.06 | 3.67 | 0.000 | 0.003** |
| ERN13 | 0.22 | 0.07 | 3.20 | 0.001 | 0.005** |
| ERN23 | 0.23 | 0.06 | 3.67 | 0.000 | 0.003** |
| ERN24 | 0.25 | 0.07 | 3.41 | 0.001 | 0.004** |
| ERN34 | 0.23 | 0.06 | 3.54 | 0.000 | 0.003** |
| *Note.* Estimates derived form a multilevel mediation analysis that tested whether youth’s network connectivity (peak-based) mediated the relationship between parent’s current psychopathology, psychiatric history and their interaction term and youth psychopathology at a 3-year follow-up. Presented are standardized coefficients (*p_uncorrected_* < .05) that represent the main effect of parent’s psychiatric history on youth psychopathology.  ERN = Emotion regulation network; *se* = Standard error; Alpha = 0.05  * *p* < .05 ** *p* < .01 | | | | | |

References

Achenbach, T. M. (2009). *Achenbach System of Empirically Based Assessment (ASEBA): Development, Findings, Theory, and Applications*. University of Vermont, Research Center of Children, Youth & Families. https://books.google.de/books?id=JMsdcgAACAAJ

Benjamini, Y., & Hochberg, Y. (1995). Controlling the False Discovery Rate: A Practical and Powerful Approach to Multiple Testing. *Journal of the Royal Statistical Society: Series B (Methodological)*, *57*(1), 289–300. https://doi.org/10.1111/j.2517-6161.1995.tb02031.x

Brett, M., Anton, J.-L., Valabregue, R., & Poline, J.-B. (2002). *MarsBaR. Region of interest analysis using an SPM toolbox [abstract]* [Computer software].

Brown, S. A., Brumback, T., Tomlinson, K., Cummins, K., Thompson, W. K., Nagel, B. J., De Bellis, M. D., Hooper, S. R., Clark, D. B., Chung, T., Hasler, B. P., Colrain, I. M., Baker, F. C., Prouty, D., Pfefferbaum, A., Sullivan, E. V., Pohl, K. M., Rohlfing, T., Nichols, B. N., … Tapert, S. F. (2015). The National Consortium on Alcohol and NeuroDevelopment in Adolescence (NCANDA): A Multisite Study of Adolescent Development and Substance Use. *Journal of Studies on Alcohol and Drugs*, *76*(6), 895–908. https://doi.org/10.15288/jsad.2015.76.895

Caballero, C., Nook, E. C., & Gee, D. G. (2023). Managing fear and anxiety in development: A framework for understanding the neurodevelopment of emotion regulation capacity and tendency. *Neuroscience & Biobehavioral Reviews*, *145*, 105002. https://doi.org/10.1016/j.neubiorev.2022.105002

Coalson, T. S., Van Essen, D. C., & Glasser, M. F. (2018). The impact of traditional neuroimaging methods on the spatial localization of cortical areas. *Proceedings of the National Academy of Sciences*, *115*(27). https://doi.org/10.1073/pnas.1801582115

Feczko, E., Conan, G., Marek, S., Tervo-Clemmens, B., Cordova, M., Doyle, O., Earl, E., Perrone, A., Sturgeon, D., Klein, R., Harman, G., Kilamovich, D., Hermosillo, R., Miranda-Dominguez, O., Adebimpe, A., Bertolero, M., Cieslak, M., Covitz, S., Hendrickson, T., … Fair, D. A. (2021). *Adolescent Brain Cognitive Development (ABCD) Community MRI Collection and Utilities* [Preprint]. Neuroscience. https://doi.org/10.1101/2021.07.09.451638

Fischl, B., Salat, D. H., Busa, E., Albert, M., Dieterich, M., Haselgrove, C., Van Der Kouwe, A., Killiany, R., Kennedy, D., Klaveness, S., Montillo, A., Makris, N., Rosen, B., & Dale, A. M. (2002). Whole Brain Segmentation. *Neuron*, *33*(3), 341–355. https://doi.org/10.1016/S0896-6273(02)00569-X

Fortin, J.-P. (2021). *neuroCombat: Harmonization of multi-site imaging data with ComBat*.

Garnefski, N., Rieffe, C., Jellesma, F., Terwogt, M. M., & Kraaij, V. (2007). Cognitive emotion regulation strategies and emotional problems in 9–11-year-old children: The development of an instrument. *European Child & Adolescent Psychiatry*, *16*(1), 1–9. https://doi.org/10.1007/s00787-006-0562-3

Gordon, E. M., Laumann, T. O., Adeyemo, B., Huckins, J. F., Kelley, W. M., & Petersen, S. E. (2016). Generation and Evaluation of a Cortical Area Parcellation from Resting-State Correlations. *Cerebral Cortex*, *26*(1), 288–303. https://doi.org/10.1093/cercor/bhu239

Gross, J. J., & John, O. P. (2003). Individual differences in two emotion regulation processes: Implications for affect, relationships, and well-being. *Journal of Personality and Social Psychology*, *85*(2), 348–362. https://doi.org/10.1037/0022-3514.85.2.348

Gullone, E., & Taffe, J. (2012). The Emotion Regulation Questionnaire for Children and Adolescents (ERQ-CA): A psychometric evaluation. *Psychological Assessment*, *24*(2), 409–417. https://doi.org/10.1037/a0025777

Morawetz, C., Riedel, M. C., Salo, T., Berboth, S., Eickhoff, S. B., Laird, A. R., & Kohn, N. (2020). Multiple large-scale neural networks underlying emotion regulation. *Neuroscience & Biobehavioral Reviews*, *116*, 382–395. https://doi.org/10.1016/j.neubiorev.2020.07.001

Rice, J. P., Reich, T., Bucholz, K. K., Neuman, R. J., Fishman, R., Rochberg, N., Hesselbrock, V. M., Nurnberger, J. I., Schuckit, M. A., & Begleiter, H. (1995). Comparison of direct interview and family history diagnoses of alcohol dependence. *Alcoholism, Clinical and Experimental Research*, *19*(4), 1018–1023. https://doi.org/10.1111/j.1530-0277.1995.tb00983.x

Silvers, J. A. (2022). Adolescence as a pivotal period for emotion regulation development. *Current Opinion in Psychology*, *44*, 258–263. https://doi.org/10.1016/j.copsyc.2021.09.023

Silvers, J. A., & Guassi Moreira, J. F. (2019). Capacity and tendency: A neuroscientific framework for the study of emotion regulation. *Neuroscience Letters*, *693*, 35–39. https://doi.org/10.1016/j.neulet.2017.09.017

Zhang, Y., Padmanabhan, A., Gross, J. J., & Menon, V. (2019). Development of Human Emotion Circuits Investigated Using a Big-Data Analytic Approach: Stability, Reliability, and Robustness. *The Journal of Neuroscience*, *39*(36), 7155–7172. https://doi.org/10.1523/JNEUROSCI.0220-19.2019
